# The dynamic expression of YAP is essential for the development of male germ cells derived from human embryonic stem cells

**DOI:** 10.1038/s41598-024-66852-x

**Published:** 2024-07-08

**Authors:** Sujittra Khampang, Chanchao Lorthongpanich, Chuti Laowtammathron, Phatchanat Klaihmon, Sukanya Meesa, Wichuda Suksomboon, Nittaya Jiamvoraphong, Pakpoom Kheolamai, Sudjit Luanpitpong, Charles A. Easley, Eisa Mahyari, Surapol Issaragrisil

**Affiliations:** 1 Siriraj Center of Excellence for Stem Cell Research, Faculty of Medicine Siriraj Hospital, Mahidol University, Bangkok, 10700 Thailand; 2 Division of Medical Genetics, Department of Obstetrics and Gynaecology, Faculty of Medicine Siriraj Hospital, Mahidol University, Bangkok, 10700 Thailand; 3 Center of Excellence in Stem Cell Research and Innovation, Faculty of Medicine, Thammasat University, Pathum Thani, 12121 Thailand; 4 Division of Neuropharmacology and Neurologic Diseases, Emory National Primate Research Center, Emory University, Atlanta, GA 30329 USA; 5 Department of Environmental Health Sciences, College of Public Health, University of Georgia, Athens, GA 30602 USA; 6 Regenerative Bioscience Center, University of Georgia, Athens, GA 30602 USA; 7 Division of Genetics, Oregon National Primate Research Center, Oregon Health and Science University, Portland, OR 97006 USA; 8 Division of Hematology, Department of Medicine, Faculty of Medicine Siriraj Hospital, Mahidol University, Bangkok, 10700 Thailand; 9 Bangkok Hematology Center, Wattanosoth Hospital, BDMS Center of Excellence for Cancer, Bangkok, 10310 Thailand

**Keywords:** YAP, Hippo signaling pathway, Spermatogonia stem cells, Spermatogenic cell differentiation, Human embryonic stem cells, Cell biology, Developmental biology, Stem cells

## Abstract

YAP plays a vital role in controlling growth and differentiation in various cell lineages. Although the expression of YAP in mice testicular and spermatogenic cells suggests its role in mammalian spermatogenesis, the role of YAP in the development of human male germ cells has not yet been determined. Using an in vitro model and a gene editing approach, we generated human spermatogonia stem cell-like cells (hSSLCs) from human embryonic stem cells (hESCs) and investigated the role of YAP in human spermatogenesis. The results showed that reducing YAP expression during the early stage of spermatogenic differentiation increased the number of PLZF^+^ hSSLCs and haploid spermatid-like cells. We also demonstrated that the up-regulation of YAP is essential for maintaining spermatogenic cell survival during the later stages of spermatogenic differentiation. The expression of YAP that deviates from this pattern results in a lower number of hSSLCs and an increased level of spermatogenic cell death. Taken together, our result demonstrates that the dynamic expression pattern of YAP is essential for human spermatogenesis. Modulating the level of YAP during human spermatogenesis could improve the production yield of male germ cells derived from hESCs, which could provide the optimization method for in vitro gametogenesis and gain insight into the application in the treatment of male infertility.

## Introduction

Spermatogenesis is a physiological process by which spermatogonia stem cells (SSCs) undergo meiotic division and differentiation into mature germ cells or sperms. The molecular mechanism underlying spermatogenesis has not been fully understood due to its complex process involving many cellular signals from both somatic and testicular germ cells that regulate the proliferation and differentiation of spermatogenic cells. The GDNF-GFRa1 pathway has been shown to regulate SSC self-renewal and maintain the spermatogenic niche^1–3^. In mammals, glial cell-derived neurotrophic factor (GDNF), secreted by Sertoli cells, binds to the cRET/GFRa1 receptor in SSCs and activates the zinc finger of promyelocytic leukemia (PLZF, also known as ZBTB16 or ZFP145) and Lin28 to maintain SSCs^1^. PLZF is an SSC-specific transcriptional factor required for the self-renewal and maintenance of SSCs^4–6^. Depletion of PLZF in SSCs causes aberrant activation of mTORC1 that results in low expression of cRET/GFRa1 receptor in response to GDNF and impaired their self-renewal capacity^6^. Furthermore, PLZF depletion also increases SSC apoptosis and dysregulates the tubular structure of the testes^5^. This evidence suggests the vital role of PLZF in maintaining normal spermatogenesis and producing healthy germ cells.

The Hippo signaling pathway is evolutionarily conserved and plays a critical role in cell proliferation and cell fate determination during various developmental processes^7^. The Yes-associated protein (YAP), a major downstream component of the Hippo signaling pathway, regulates cell growth, survival, and proliferation in many tissues and organs^8^. When the Hippo signaling pathway is active, the transcriptional regulator 1 consisting of YAP and WW (WWTR1 or TAZ) is phosphorylated, retained in the cytoplasmic compartment, and then targeted for destruction by proteasomes. On the other hand, when the Hippo signaling pathway is inactive, unphosphorylated YAP and TAZ translocate to the nucleus and form complexes with transcription factors to regulate gene expression^9^. While the expression of YAP/TAZ has previously been found in both somatic cells^10^ and spermatogenic cells in animal testes^10–12^, little is known about the role of the Hippo signaling pathway in the development of the mammalian germline. Although a previous study using the YAP knockout mouse model showed that the loss of YAP in germ cells did not significantly affect spermatogenesis in male mice^11^, a more recent in vitro model using pluripotent epiblast cells has identified YAP as a crucial mediator for germ cell establishment through the mesodermal WNT signaling pathway^13^. Another recent report showed that inactivation of YAP/TAZ in gonadotropes also resulted in the increased plasma levels of follicle-stimulating hormone (FSH), luteinizing hormone (LH), and testosterone^14^, and increased testicular weight and sperm density in male mice^14^. Furthermore, TAZ deficiency negatively affected the testicular structure, increased the level of apoptosis and senescence in spermatogenic cells and Leydig cells resulting in a decrease in testosterone secretion, low sperm production and impaired fertility^15,16^. Collectively, these data suggest that the expression of YAP/TAZ affects mouse spermatogenesis. However, the role of the Hippo-YAP signaling pathway in human spermatogenesis has not yet been elucidated.

Pluripotent stem cells (PSCs, including embryonic stem cells (ESCs) and induced pluripotent stem cells (iPSCs), can self-renew, and differentiate into all types of cells in the body, including human germ cell-like cells (GCLCs)^17–21^. In mice, the success of in vitro gametogenesis has been demonstrated by the production of functional female^22^ and male^23^ germ cells from PSCs that could generate healthy offsprings. Furthermore, female gametes (mature oocytes) derived from trans-differentiation of the male XY cells could be fertilized and produce healthy progenies^24^. These studies clearly demonstrate that functional germ cells can be derived from mouse pluripotent stem cells. In a non-human primate, a recent report by Khampang et al. also showed that round haploid spermatids derived from monkey ESCs could fertilize monkey oocytes after intra-cytoplasmic sperm injection (ICSI), resulting in viable blastocysts^25^. In humans, although the in vitro production of human germ cells from PSCs has been reported, access to fertilization and transplantation remains ethically challenged. Xenotransplantation of undifferentiated human iPSC directly into murine seminiferous tubules found that human iPSCs could differentiate extensively into germ cell-like cells (GCLC) that were located near the basement membrane of mice seminiferous tubules. These GCLCs exhibited a morphology similar to that of fetal germ cells and expressed primordial germ cell markers^26^. Human PSC-derived hGCLCs have also been shown to proliferate and colonize the basal membrane of seminiferous tubules of genetically infertile nude mice^20,27^. These studies demonstrate the ability of hGCLCs to engraft and further develop in the microenvironment of mouse testes which are quite different from that of human testes.

Currently, hSSLCs derived from PSCs have been established as a model for studying male germ cell development, and are considered a potential source of spermatogenic cells for treating infertility in both humans and animals^18,28–31^. Taking advantage of this recently established in vitro spermatogenesis system, we investigated the regulatory roles of YAP during human spermatogenesis and the subsequent development of haploid germ cells derived from hESCs. We believe that the knowledge gained from this study could be used to improve the efficiency of in vitro spermatogenesis to generate a sufficient number of human male germ cells for future applications.

## Materials and methods

### In vitro derivation of spermatogenic cells from hESCs

hESCs (MUSIe002-A) derived from a male donor^32^ were maintained in a T-25 culture flask pre-coated with Matrigel (Corning, Inc., Corning, NY, USA) in NutriStem™ Medium (Biological Industries, Cromwell, CT, USA). The cells were cultured under hypoxic condition (5% CO_2_, 5%O_2_) to reach 80% confluence before being passaged using Versene™ (Thermo Fisher Scientific) and replated in each well of a 12-well plate and kept in NutriStem™ Medium for 2 days to reach 80% confluence before being cultured in spermatogenic cell differentiation (SSC) medium to initiate the differentiation. The MUSIe002-A cells were differentiated into spermatogenic cells according to the protocols described previously^18,21^. Briefly, hESCs were cultured in SSC medium, which is alpha-MEM supplemented with 0.2% bovine serum albumin (Sigma), 2 mM L-glutamine (Gibco), 0.2% chemically defined lipid concentrate (Gibco), 200 ug/ml vitamin C (Sigma), 1 × Insulin-Transferrin-Selenium-X (Gibco), 10 mM HEPES (Gibco), 1 ng/ml human basic fibroblast growth factor (bFGF: Prepotech), 20 ng/ml recombinant human GDNF (R&D system), 100 U/ml penicillin, and 100 ug/ml streptomycin. Before use, the medium was gassed with a blood gas mixture (5% CO_2_, 5%O2, 90% N_2_ gas). The medium was replaced every day for 12 days. Differentiated cells were collected for analysis on days 5, 10, and 12 and compared with non-differentiated hESCs on day 0. Bright-field imaging was recorded using the Nikon Eclipse Ti-U inverted microscope (Nikon Corporation, Tokyo, Japan).

### Generation of the YAP knockdown cell line for in vitro spermatogenic differentiation

To create a YAP knockdown cell line to study the effect of YAP during human spermatogenesis in vitro, MUSIe002-A hESCs were transduced with the CRISPR/Cas9 plasmid targeting the *YAP1* locus to establish YAP knockdown hESCs. Briefly, CRISPR lentiviral vector with insert hCas9: T2A: Neo containing single guide RNA targeting *YAP1* (VB190408-1213vaj; VectorBuilder, CA, USA) was transfected into HEK293T packaging cells using Lipofectamine 3000 (Life Technologies, Carlsbad, CA, USA) for 24 h. After transfection, the cells were cultured in DMEM (Gibco) supplemented with 10% FBS for two days. The culture medium containing viral particles was then collected and concentrated with an Amicon Ultra-15 centrifugal filter tube (Merck Millipore) by centrifugation at 4000 g for 30 min at 4 °C. The concentrated virus was incubated with 4 × 10^5^ hESCs in NutriStem™ Medium containing 5 μg/ml polybrene (Sigma-Aldrich) for 24 h. The transfected hESCs were then selected with 2 μg puromycin. Healthy hESC colonies were dissociated into single cells and subjected to single-cell colony formation in a 96-well culture plate. After 5–7 days of culture, the emerging colonies were picked up, expanded, and harvested to determine their YAP expression by Western Blot. The YAP knockdown hESCs, MUSIe002-A-1, were successfully established^33^ and later referred to as YAP-KD cells in this manuscript.

To further inhibit YAP expression, the retroviral shRNA plasmid (pSuper-Retro-puro) targeting *YAP*, a kind gift from Dr. Siew Wee CHAN, IMCB, A* STAR Laboratory, Singapore, was transduced into the YAP-KD cells to establish the YAP double knockdown (YAP-DKD) cells. For the production of retroviral particles, shRNA plasmids were transfected into a Platinum-A retroviral packaging cell. The viral particles containing the plasmid were then transduced into the YAP-KD cells, and a single clone selection was performed. The established YAP-KD and YAP-DKD cells were then subjected to in vitro spermatogenic cell differentiation compared to wild-type hESCs.

### Immunofluorescent Staining

Cells were washed twice with PBS, fixed with 4% paraformaldehyde (PFA) for 30 min, and permeabilized with 0.5% Triton X-100 for 30 min. For cells collected from fluorescent activated cell sorting (FACS), the sorted cells were cytospun onto a glass slide at 1000 rpm for 5 min before being fixed and permeabilized. Fixed cells were then blocked with 10% FBS in PBS for 1 h and incubated at 4 °C overnight with primary antibodies against the following human proteins: VASA/DDX4 (ab13840, Abcam), CD90/THY1 (ab23894, Abcam), GPR125 (ab51705, Abcam), PIWIL (ab12337, Abcam), PLZF (MAB2944, R&D Systems), Acrosin (PA5-99,580, Thermo Fisher), and TNP1 (ab73135, Abcam). After removing the primary antibody, the cells were washed with PBS and incubated with appropriate secondary antibodies (Thermo Fisher) for 1 h. Finally, the cells were counterstained with Hoechst 33,342 (Thermo Scientific, MA, USA) for 10 min at room temperature and examined with a Nikon Eclipse Ti-U inverted fluorescence microscope (Nikon Corporation, Tokyo, Japan).

### Flow cytometry and fluorescent activated cell sorting (FACS) analysis

Cells were dissociated into single cells using TryPLE (Gibco) and fixed with 2% PFA for 20 min. The fixed cells were then permeabilized with 0.5% Triton X for 30 min and blocked in 0.2% BSA in PBS for 30 min. The cells were then incubated with a primary antibody against human PLZF (2.5 µg/10^6^ cells) for 1 h before incubation with an appropriate secondary antibody for another 1 h at room temperature. For flow cytometry, cells were incubated with FITC-conjugated anti-human CD90 (Thy1; 5ul/10^6^ cells), Alexa Fluor 700-conjugated anti-human PLZF (0.25–1 µg/10^6^ cells) and APC-conjugated anti-human CD117 (c-Kit; 5ul/10^6^ cells) for 30 min prior to flow cytometry analysis.

To analyze DNA content and cell cycle, single cells were stained with propidium iodide (PI) using the cell cycle assay; BD CycleTest™ Plus DNA reagent kit (BD Biosciences) according to the manufacturer’s instructions. The samples were subjected to flow cytometry analysis using a FACS Canto cytometer (BD Biosciences). To harvest the haploid cells, the dissociated cells were stained with 15 mg/ml Hoechst 33,342 (Thermo Scientific, MA, USA) in SSC medium at 37 °C for 20 min and sorted by BD Aria Fusion cell sorter. The sorted cells were then centrifuged and washed with PBS before further analysis.

### Fluorescent in situ hybridization (FISH)

Cells were sent to the Prenatal Cytogenetic Laboratory Faculty of Medicine Siriraj Hospital, Mahidol University, for fluorescence in situ hybridization (FISH). Briefly, single cells were incubated in 2 ml of pre-warmed 0.075 M KCl for 20 min at 37 °C. Cells were then fixed with fresh cold Carnoy fixative (3:1 methanol to glacial acetic acid) and centrifuged at 1,400 rpm for 7 min. The cell suspension was dropped onto the slide and dried overnight at room temperature. The dried slide was washed with 70%, 80%, and 100% ethanol and air dried at room temperature. FISH was carried out using locus-specific probes for chromosome 13, chromosome 21, centromeric region of X chromosome, and SRY region of Y chromosome (Vysis, Abbott, USA) following the manufacturer’s instructions. After probe hybridization, the slides were washed, air-dried, and counterstained with DAPI II (Abbott, USA). Fifty interphase nuclei were scored and captured by a fluorescent microscope.

### Analysis of single-cell RNA sequencing data

To represent the expression profiles of YAP and other markers in human testes, single-cell RNA sequencing results were obtained using the Human Infertility Testis Atlas (HISTA) web-tool (https://conradlab.shinyapps.io/HISTA/). As previously described^34^, the HISTA gene expression data is a batch-cleaned aggregated data set of human testes scRNA-seq from multiple sources, comprising cells from adult and juvenile controls, as well as several infertile cases, including two patients with Klinefelter syndrome. However, the data shown in this manuscript were re-analyzed from the previous publication^26^ and include only the data set of healthy adult donors (N = 6). No humans were involved in this study. Briefly, these data sets were integrated and analyzed using an unsupervised soft-clustering approach that clusters genes and cells into latent “components”, which are then manually interpreted and separated into “technical noise” and “signal”.

### Western blot analysis

Cell pellets were lysed using lysis buffer (1 × RIPA; Cell Signaling Technology, Danvers, MA, USA) supplemented with protease inhibitor (Roche Life Science) at 4 °C for 30 min. Protein concentrations were measured using the BCA protein assay (Pierce Biotechnology, Rockford, IL, USA). The isolated proteins were then separated on SDS/polyacrylamide gels, transferred onto PVDF membranes (Merck Millipore), and blocked with 5% non-fat skim milk (Sigma Andrich) at room temperature for 1 h. The membranes were then incubated with appropriate primary antibodies at 4 °C overnight before incubation with the peroxidase conjugated secondary antibody at room temperature for 1 h. The protein levels were then detected by an enhanced chemiluminescence detection system using an ImageQuant LAS digital imager (GE Healthcare, Pittsburgh, PA, USA). Antibodies and concentrations used in the experiments were listed in Supplemental Table S1.

### Quantitative real-time PCR (qRT-PCR)

Total RNA was isolated from cells using TRIzol (Invitrogen, Carlsbad, CA, USA). Two micrograms of isolated RNA were converted to cDNA using the RevertAid First Strand cDNA synthesis kit (Thermo Fisher, Waltham, MA, USA). Real-time PCR reactions were performed on a CFX384 Touch Real-Time PCR Detection System (Bio-Rad Laboratories, Hercules, CA, USA) using the SYBR™ Green master mix (Applied Biosystems, Foster City, CA, USA). *GAPDH* was used as a housekeeping gene to normalize the data. Primers are listed in Supplemental Table S1.

### Ethics approval and consent to participate

The study protocol was approved by Siriraj Institutional Review Board, Faculty of Medicine, Siriraj Hospital (SIRB), Mahidol University (Si338/2013). The protocols used in this study complied with the principles set forth in the Declaration of Helsinki, the Belmont Report, the CIOMS Guidelines, and the ICH-GCP. All experiments were performed under the guidelines and regulations of SIRB, Mahidol University. Informed consent was obtained from all subjects and/or their legal guardian.

### Statistical analysis

Data are presented as mean ± standard deviation (SD) of at least three independent experiments. The unpaired Student’s t-test and One-way ANOVA was performed to determine significant differences between two and three experimental groups respectively using Prism9 software. A *P*-value less than 0.05 was considered statistically significant. The Wilcox rank test was used for analysis of single cell RNA seq data.

## Results

### Derivation of spermatogenic cells from human embryonic stem cells (hESCs)

**Figure 1:**
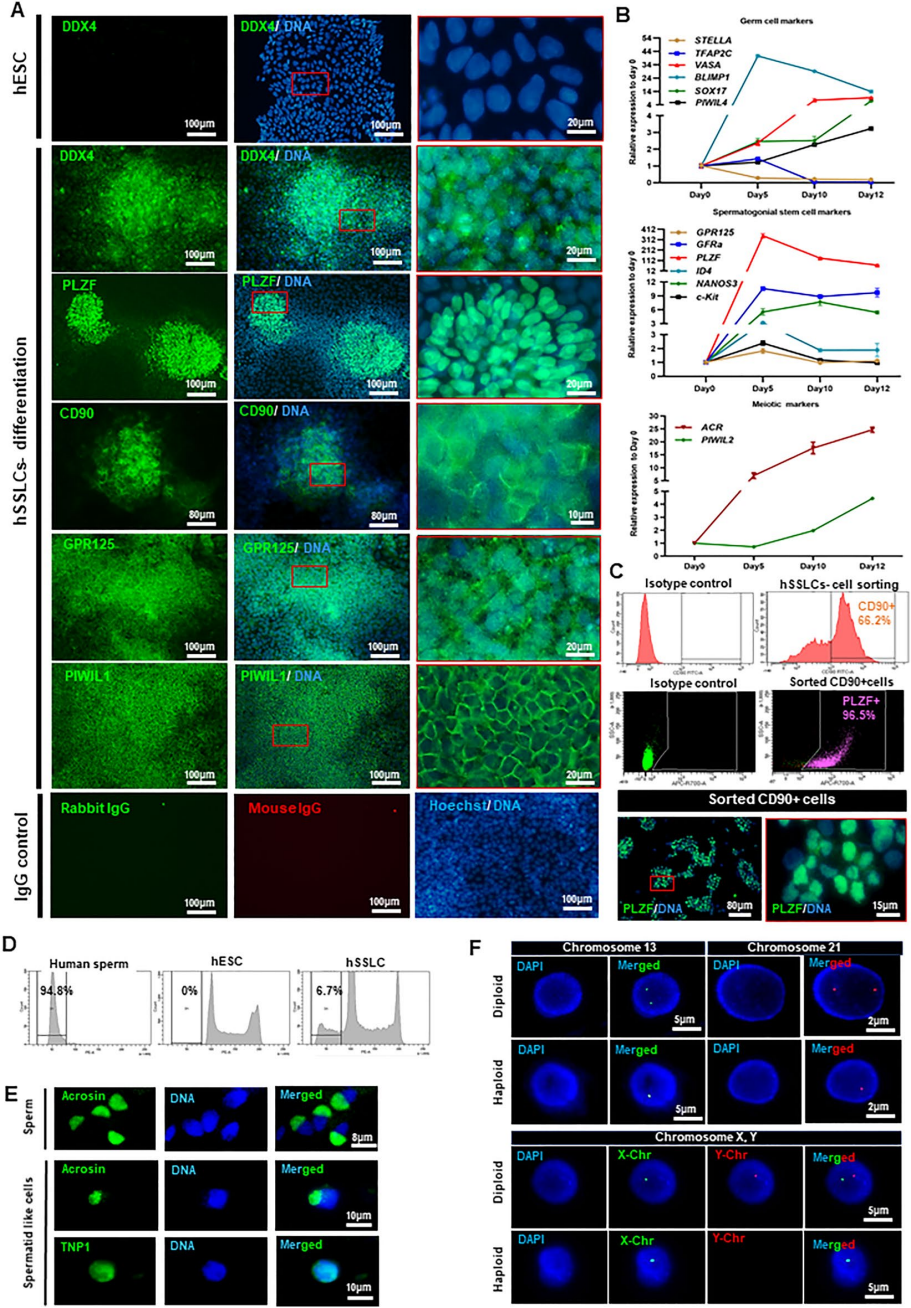
Generation of hESC-derived spermatogenic cells. (**A**) Representative immunofluorescence micrographs show the expression of germ cell markers VASA, the hSSC markers PLZF, CD90, GPR125, and the spermatocyte marker PIWIL1 in hSSLC. Non-differentiated hESCs on culture day 0 and IgG staining served as a control. Scale bar: 100 μm in the first and middle panel, 20 μm in the last panel. The micrographs shown in the last column are the zoomed-in of the area indicated in red box (**B**). The graphs show the expression levels of the early germ cell markers (*STELLA*, *TFAP2C, BLIMP1, SOX17, PIWIL4* and *VASA),* hSSC markers (*PLZF*, *GFRA*, *GPR125, ID4, NANOS3 and c-Kit*)*,* spermatocyte (*PIWIL2*) and spermatid marker (*ACR*) during spermatogenic differentiation of hESCs relative to those on culture day 0. Data are presented as mean ± SD of 3 experiments. (**C**) Expression of CD90^+^ cells (top row), PLZF^+^ cells in the sorted CD90^+^ cell population determined by flow cytometry (middle row) and immunofluorescent staining (bottom row). The picture in the right panel showed the zoomed-in area indicated in red. (**D**) Analysis of DNA content by flow cytometry shows the percentages of hESC-derived haploid spermatid-like cells at the end of spermatogenic differentiation. Non-differentiated hESCs on culture day 0 serves as a negative control, while mature human sperm serves as a positive control. (**E**) Representative immunofluorescence micrographs show the expression of the haploid germ cell marker Acrosin in human sperm cells (top row), Acrosin and TNP1 in hESC-derived spermatid-like cells. (F) Representative micrographs show a single copy of chromosome 13, 21, X and Y chromosome in hESC-derived spermatid-like cells determined by fluorescent in situ hybridization (FISH). Non-differentiated hESCs, which served as a control, had two copies of these chromosomes.

To determine the spermatogenic differentiation potential of non-manipulated hESCs, MUSIe002-A were subjected to a spermatogenic differentiation procedure. At the end of culture, the hESC-derived spermatogenic cells expressed several human spermatogenic marker proteins, including the pan-germ cell marker (VASA), spermatogonia stem cell markers (PLZF, CD90 and GPR125), and a spermatocyte marker (PIWIL1) (Fig. 1A) compared to non-differentiate hESCs on culture day 0 (Fig. S1). In accordance with their protein expression, hESC-derived spermatogenic cells were subjected to transcriptional analysis by qRT-PCR. Up-regulation of *SOX17* and *TFAP2C* was observed, which indicated the development toward germ cell lineages^35^. Furthermore, an early PCG marker *BLIMP1,* the post-migratory gonocyte *PIWIL4* and the germline-specific gene *VASA* were up-regulated on day 5 and were maintained to the end of culture. Spermatogonia markers, including *PLZF, GFRA, and NANOS3,* were found to be highly expressed on day 5 and to remain throughout the culture period, while expressions of *GRP125*, *ID4,* and *c-Kit* were found to be upregulated on day 5 after induction (Fig. 1B). Furthermore, the expression levels of the meiotic marker *PIWIL2*, and the post-meiotic haploid marker *ACR,* in differentiated hESCs also increased steadily and reached their highest levels at the end of culture (Fig. 1B).

Next, we determined the percentage of hESC-derived hSSLCs after spermatogenic differentiation using the cell surface marker CD90, which has previously been used to identify SSLC in human and mouse models^20,36^. The results show that 66% of differentiated hESCs expressed CD90 (Fig. 1C; top row). We also found that more than 90% of the CD90 + hSSLCs also co-expressed PLZF, another widely used SSLC marker, as determined by flow-cytometry and immunofluorescent staining (Fig. 1C; middle and bottom rows, respectively). These results confirm that hESC-derived hSSLCs are present in the culture after spermatogenic differentiation and could be identified with a combination of CD90 and PLZF markers.

To determine whether the differentiated hESCs generated any haploid spermatid-like cells, a DNA content analysis was performed. The results show that 6.7 ± 0.72% of the differentiated hESCs are haploid cells, while no haploid cell was detected in non-differentiated hESCs (Figs. 1D and S2). The haploid spermatid-like cells derived from hESCs expressed human spermatid markers, Acrosin and TNP1 (Figs. 1E and S2). Furthermore, fluorescent in situ hybridization (FISH) analysis confirmed that our sorted haploid spermatid-like cell derived from hESCs has a single copy of chromosome 13, 21 and sex chromosome compared to two copies of the same chromosomes in non-differentiated hESCs (Fig. 1F). These results demonstrated that the spermatogenic differentiation procedure used in this study successfully generated haploid spermatid-like cells from hESCs. The pattern of marker expression during in vitro spermatogenic differentiation closely matched that of human testicular germ cells that underwent in vivo spermatogenesis (Supplementary Fig. S3A–G)^34^. Collectively, these results confirm that we could derive male germ cells from hESCs in vitro using the protocol previously described by Easley et al.^21^. Next, we determine whether Hippo/YAP is expressed in human testicular germ cells. Analysis of scRNA sequencing data showed YAP expression during spermatogenesis (Supplementary Fig. S3H,I). Like *PLZF*, the expression level of *YAP* was higher in undifferentiated human spermatogonia compared to the later stages of human spermatogenic differentiation (Supplementary Fig. S3H,I). This result suggested that *YAP* might play a crucial role during the early stages of human spermatogenesis and could be involved in the expression of the undifferentiated spermatogonia marker *PLZF*.

### YAP depletion at the beginning of spermatogenic differentiation promotes germline specification of hESCs

**Figure 2:**
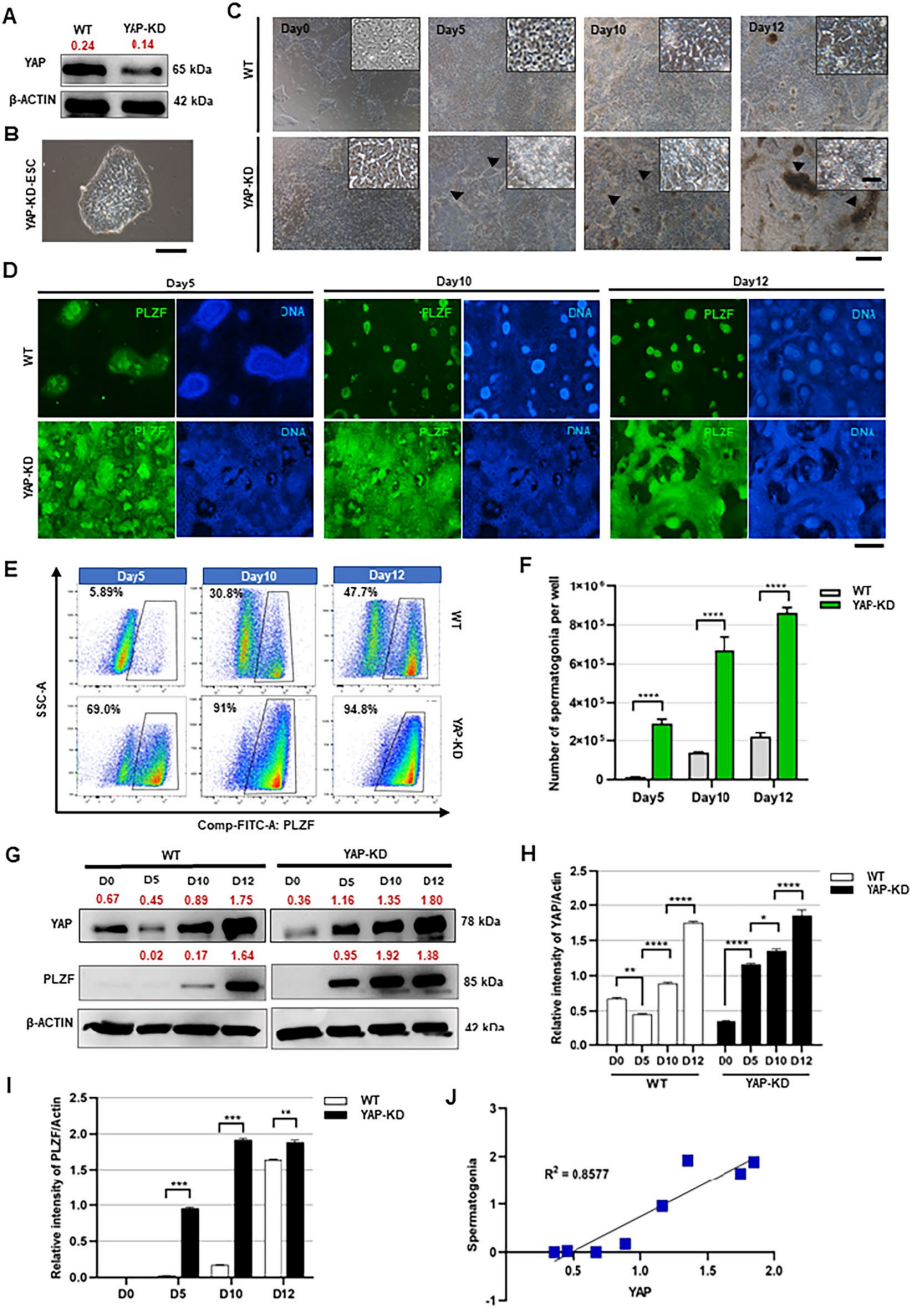
Spermatogenic differentiation of YAP knockdown hESCs (YAP-KD cells). (**A**) Western blot shows the level of YAP relative to β-ACTIN in YAP-KD cells compared to wild-type hESCs (WT). (**B**) Representative micrograph showing the morphology of a non-differentiated YAP-KD cell colony. Scale bar: 200 μm. (**C**) Representative micrographs show the morphology of differentiated YAP-KD cells during their spermatogenic differentiation. Wild-type hESCs cultured under the same conditions (WT) serve as controls. Scale bar: 200 μm in micrographs, 50 μm in zoomed areas. (**D**) Representative immunofluorescence micrographs show the expression of the SSC marker PLZF in YAP-KD cells during their spermatogenic differentiation compared to WT cells. Scale bar: 200 μm. (**E**) Representative dot plots show the percentages of PLZF^+^ hSSLCs in YAP-KD cells during their spermatogenic differentiation compared to those of WT cells. (**F**) Graphs show the number of PLZF^+^ hSSLCs derived from YAP-KD cells during their spermatogenic differentiation compared to those of WT cells. Data are presented as mean ± SD from 3 experiments. ****P < 0.0001. (**G**) Western blot shows the levels of the YAP and PLZF proteins relative to β-ACTIN in YAP-KD cells during their spermatogenic differentiation compared to those of WT cells. (**H**) Graphs show the expression level of YAP protein relative to β-ACTIN in YAP-KD cells during their spermatogenic differentiation compared to that of WT cells. Data are presented as mean ± SD of 3 experiments. *P < 0.05, **P < 0.01, ****P < 0.0001. (**I**) Graphs show the expression level of PLZF protein relative to β-ACTIN in YAP-KD cells during their spermatogenic differentiation compared to that of WT cells. Data are presented as mean ± SD of 3 experiments. **P < 0.01, ***P < 0.001. (J) Correlation analysis between YAP protein levels and hESC-derived PLZF^+^ hSSC numbers during spermatogenic differentiation of WT and YAP-KD cells. The uncropped gels associated with Fig. 2A are shown in Supplementary Fig. S8.

To investigate the role of *YAP* during human spermatogenesis, the isogenic YAP-depleted hESCs (YAP-KD cells) were established by transducing hESCs (MUSIe002-A cells) with the *YAP*-targeted CRISPR/Cas9 plasmid^33^. Western blot analysis showed a 50% reduction in the YAP level in YAP-KD cells compared to their wild-type control (WT cells) (Fig. 2A). The YAP-KD cells retained a typical morphology of non-differentiated wild-type hESCs even after being expanded for more than 25 passages (Figs. 2B and S4). After spermatogenic differentiation, the YAP-KD cells generated several clusters of cells, which increased in size and number toward the end of the culture (indicated by black arrows in Figs. 2C and S5). These cell clusters were also observed in differentiated WT cells, but their number was less than in the differentiated YAP-KD cells (Figs. 2C and S5).

YAP protein is highly expressed in spermatogonia residing in testicular germ cells^10,11^. Similar to WT cells, differentiated YAP-KD cells highly expressed PLZF, a spermatogonia marker. Immunofluorescence shows that the level of PLZF expression in differentiated YAP-KD cells increased steadily throughout the entire culture period and was much higher than that of WT cells (Fig. 2D). Consistent with the level of PLZF expression, the percentages of PLZF-expressing hSSLCs (PLZF^+^ cells) derived from the differentiated YAP-KD cells increased steadily throughout the differentiation period and were 2–3 times higher than those derived from WT cells throughout the culture period (Figs. 2E and S6). Consistent with these data, when the absolute number of PLZF^+^ cells was determined by BD Truecount™, the YAP-KD cell also generated a higher number of PLZF^+^ cells than the WT cells, resulting in an approximately threefold increase in the number of PLZF^+^ cells at the end of the culture (8.61 × 10^5^cells vs 2.19 × 10^5^ cells on culture day 12) (Fig. 2F). This result indicated that the depletion of YAP in hESCs enhances their spermatogenic differentiation toward PLZF^+^ hSSLCs.

Next, we quantified the expression levels of YAP and PLZF proteins at different time points during the spermatogenic differentiation of YAP-KD cells compared to WT cells. The expression level of YAP in WT cells was initially downregulated on culture day 5, bounced back on culture day 10, and continued to increase towards the end of the culture (Fig. 2G,H). Interestingly, YAP-KD cells, whose YAP expression was 50% lower than that of WT cells at the beginning of culture, rapidly increased their YAP expression on culture day 5 (Fig. 2G,H). Similar to WT cells, the expression level of YAP in YAP-KD cells continued to increase toward the end of the culture. At the end of the culture (day 12), the expression level of YAP in YAP-KD cells is similar to that of WT cells. However, unlike WT cells, down-regulation of YAP during the early phase of spermatogenic differentiation (day 5) was not observed in differentiated YAP-KD cells (Fig. 2G,H).

The up-regulation of YAP in WT and YAP-KD cells coincided with the expression of PLZF, whose level also increased steadily towards the end of the culture (Fig. 2G,I). However, the differentiated YAP-KD cells expressed PLZF earlier than WT cells (day 5 vs day 10), and their levels of PLZF were significantly higher than those of WT cells throughout the culture period (Fig. 2G,I).

Furthermore, the level of YAP protein in both WT and YAP-KD cells was positively correlated with the expression level of PLZF^+^ hSSLCs generated during their spermatogenic differentiation (R^2^ = 0.8577, *P* = 0.001) (Fig. 2J). These results suggested that depletion of YAP at the beginning of spermatogenic differentiation leads to higher and earlier expression of PLZF, resulting in a higher number of hESC-derived hSSLCs.

### YAP depletion at the beginning of spermatogenic differentiation accelerates the maturation of hESC-derived hSSLCs to haploid spermatid-like cells

**Figure 3:**
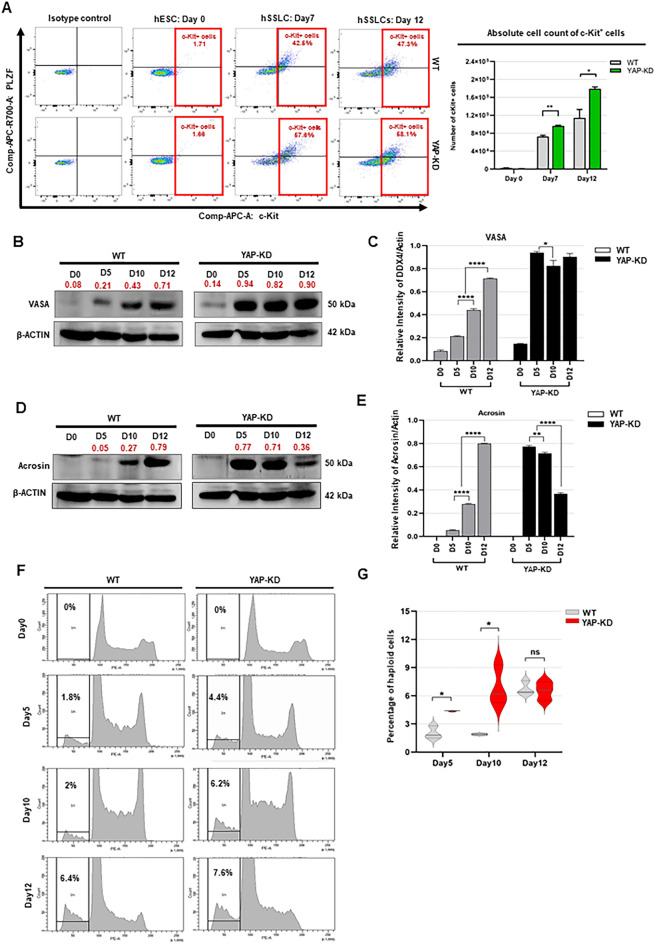
Derivation of haploid spermatid-like cells from YAP-KD cells. (**A**) Representative dot plots show the percentages of c-Kit^+^ cells in differentiated WT and YAP-KD cells during their spermatogenic differentiation (left). The graph shows the absolute number of c-Kit^+^ differentiated spermatogonia derived from YAP-KD cells during their spermatogenic differentiation compared to those of WT cells (right). Data are presented as mean ± SEM from 3 replicates. *P < 0.05, **P < 0.01 (**B**) Western blot shows the levels of VASA proteins relative to β-ACTIN in YAP-KD cells during their spermatogenic differentiation compared to those of WT cells. (**C**) Graphs show the expression level of VASA protein relative to β-ACTIN in YAP-KD cells during their spermatogenic differentiation compared to that of WT cells. Data are presented as mean ± SD of 3 experiments. *P < 0.05, ****P < 0.0001. (**D**) Western blot shows the levels of Acrosin proteins relative to β-ACTIN in YAP-KD cells during their spermatogenic differentiation compared to those of WT cells. (**E**) Graphs show the expression level of Acrosin protein relative to β-ACTIN in YAP-KD cells during their spermatogenic differentiation compared to that of WT cells. Data are presented as mean ± SD of 3 experiments. **P < 0.01, ****P < 0.0001. (**F**) Analysis of DNA content by flow cytometry shows the percentages of haploid spermatid-like cells derived from YAP-KD cells during their spermatogenic differentiation compared to those of WT cells. (**G**) The graphs show the percentages of haploid spermatid-like cells derived from YAP-KD cells during their spermatogenic differentiation compared to those of WT cells. Data are presented as mean ± SD of 3 experiments. *P < 0.05. The uncropped gels associated with Fig. 3A,C are shown in Supplementary Fig. S9.

As shown above, early depletion of YAP in hESCs increased the percentage and absolute number of hSSLCs during spermatogenic differentiation. Next, we determine whether hESC-derived hSSLCs express c-Kit, a differentiated spermatogonia marker. We performed a flow cytometric analysis for the expression of c-Kit in hSSLCs derived from WT and YAP-KD cells at different time points during their spermatogenic differentiation. The results showed that both the percentage and the absolute number of c-Kit+ differentiated spermatogonial cells were up-regulated in the WT and YAP-KD groups as differentiation progressed towards the end of the culture. In which the percentages and absolute numbers of c-Kit+ cells in the YAP-KD groups are significantly higher than those of WT on days 7 and 12 of culture (Fig. 3A). This pattern of c-kit expression is similar to that of PLZF expression and therefore confirms that YAP depletion increases the spermatogenic differentiation of hESCs.

We then investigated the effect of YAP depletion during the early stages of spermatogenic differentiation by measuring the expression level of VASA, an early germ cell marker. We found that YAP-KD cells expressed a significantly higher level of VASA protein than WT cells throughout the entire culture period (Fig. 3B,C).

Next, we investigated the effect of YAP depletion during the later stages of spermatogenic differentiation by measuring the expression level of Acrosin, a specific marker of haploid spermatids, in differentiated YAP-KD cells. Similar to PLZF, differentiated YAP-KD cells up-regulated their acrosin expression earlier than WT cells (day 5 vs day 10) and their acrosin levels were significantly higher than those of WT cells on day 5 and day 10 of culture (Fig. 3D,E). Consistent with the level of acrosin expression, the percentages of haploid spermatid cells in differentiated YAP-KD cells were significantly higher than those of their wild-type counterparts on day 5 (4.4 ± 0.05% vs. 1.8 ± 0.68%) and day 10 of culture (6.2 ± 2.09% vs. 2 ± 0.10%) (Fig. 3F,G). These results suggest that YAP depletion at the beginning of spermatogenic differentiation could accelerate the progression of hESC-derived hSSLCs into haploid spermatid-like cells. However, we found that acrosin + spermatid-like cells could not be maintained in culture, since the level of acrosin in differentiated YAP-KD cells on day 12 of culture was lower than on day 10 (Fig. 3D).

### Increasing YAP expression during the later stages of spermatogenic differentiation is crucial for the survival and maturation of hESC-derived hSSLCs

Due to the heterozygous nature of the mutated *YAP* gene in our YAP-KD cells, up-regulation of YAP expression was observed during the later stages of spermatogenic differentiation (days 5–12) (Fig. 2G,H). This pattern of YAP expression in YAP-KD cells suggests that although YAP depletion at the beginning of spermatogenic differentiation appears to promote the germline specification of hESCs, up-regulation of YAP during the later stages of spermatogenic differentiation could be crucial for the expression of germ cell markers and the derivation of both hSSLCs and haploid spermatid-like cells.

**Figure 4:**
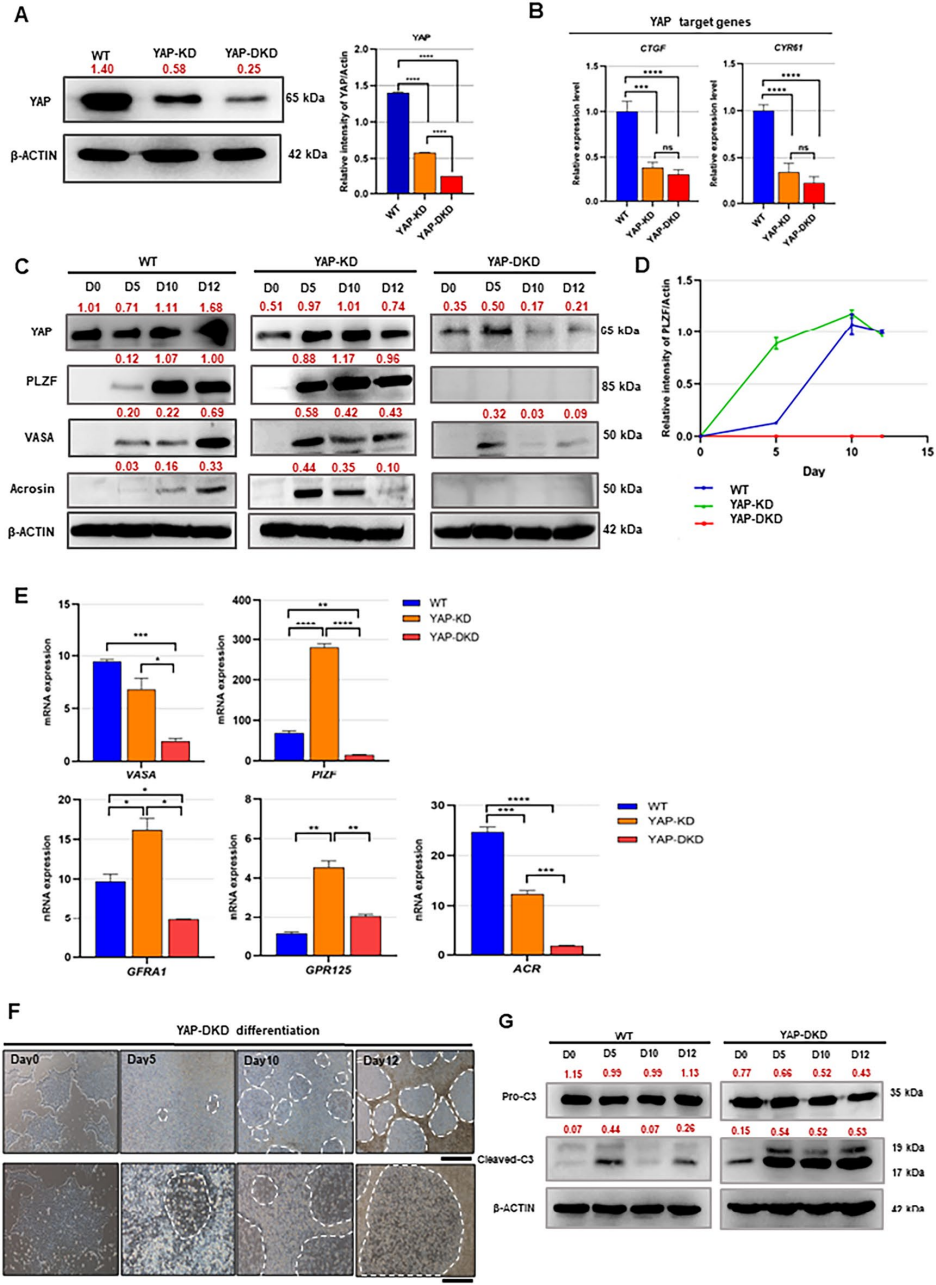
Spermatogenic differentiation of YAP double knockdown hESCs (YAP-DKD cells) (**A**). Western blot shows the level of YAP relative to β-ACTIN in YAP-DKD cells compared to that of YAP-KD cells and wild-type hESCs (WT). ****P < 0.0001. (**B**) Graphs show the expression levels of YAP target genes, *CTGF* and *CYR61*, in YAP-DKD cells compared to those of YAP-KD cells and WT. Data are presented as mean ± SD of 3 experiments. **P < 0.01, ***P < 0.001. (**C**) Western blot shows YAP, PLZF, VASA and acrosin protein levels relative to β-ACTIN in WT, YAP-KD, and YAP-DKD cells during their spermatogenic differentiation. (**D**) Graphs show the level of PLZF protein relative to β-ACTIN in YAP-DKD cells during their spermatogenic differentiation compared to YAP-KD cells and WT. (**E**) Graphs show the expression levels of spermatogenic cell markers (*VASA, PLZF, GFRA1, GPR125 and ACR)* during spermatogenic differentiation of YAP-DKD, compared to those of YAP-KD cells and WT. Data are presented as means ± SD of 3 experiments. *P < 0.05, **P < 0.01, ***P < 0.001, ****P < 0.0001. (**F**) Representative micrographs show the morphology of YAP-DKD cells during their spermatogenic differentiation. White dots indicate areas of dead cells. Scale bar: 200 μm for the upper panel and 100 μm for the lower panel. (**G**) Western blot shows the level of the apoptotic marker cleaved Caspase3 relative to β-ACTIN in WT and YAP-DKD cells during their spermatogenic differentiation. The uncropped membranes associated with this figure are provided in Supplementary Fig. S10.

To prevent the up-regulation of YAP in YAP-KD cells during the later stages of spermatogenic differentiation, a short hairpin RNA (shRNA) targeting the YAP transcript^37^ was transfected into YAP-KD cells to establish a YAP double knockdown hESCs (YAP-DKD cells). YAP-DKD cells showed a further reduction in YAP (Fig. 4A; Supplementary Table S2) and YAP target genes, *CTGF* and *CYR61*, compared to YAP-KD cells (Fig. 4B). Like YAP-KD cells, YAP-DKD cells exhibited a typical morphology of non-differentiated wild-type hESCs (Supplementary Fig. S4).

When subjected to spermatogenic differentiation, different expression patterns of YAP and early germ cell markers of each cell type were observed. Unlike YAP-KD cells, the level of YAP expression in YAP-DKD cells was barely detected on day 10 and day 12 of culture (Fig. 4C). This result confirmed that the shRNA successfully prevented the up-regulation of YAP in YAP-DKD cells during the later stages of their spermatogenic differentiation. Further analysis found that constitutive depletion of YAP in YAP-DKD inhibited the expression of the early germ cell marker VASA (Fig. 4C), the SSC marker PLZF (Fig. 4C,D), and the spermatid marker acrosin (Fig. 4C). These results suggest that the up-regulation of YAP level during the later stages of spermatogenesis might be critical for the expression of germ cell markers and the derivation of hSSLCs and haploid spermatid-like cells from hESCs.

Consistent with the protein expression study, differentiated YAP-DKD cells expressed lower transcript levels of germ cell marker (*VASA)*, hSSC marker genes (*PLZF*, *GFRA1,* and *GPR125*), and meiotic germ cell markers (*ACR*) compared to WT (Fig. 4E). In contrast, YAP-KD cells, whose YAP was depleted only at the beginning of their spermatogenic differentiation, expressed higher levels of hSSC markers*, PLZF, GPR125,* and *GFRA1* compared to WT cells (Fig. 4E). Taken together, these results suggest that depleting YAP at the beginning of spermatogenic differentiation promotes germline specification of hESCs, and increasing the level of YAP during the later stages of spermatogenic differentiation is critical for the derivation and survival of hESC-derived hSSLCs and haploid spermatid-like cells.

Furthermore, we also found that the differentiated YAP-DKD cells contained several large areas of dead cells (dotted areas in Figs. 4F and S5). Consistent with this, western blots also showed a high level of cleaved-caspase 3 in YAP-DKD cells at the later stages of spermatogenic differentiation (D5-12) compared to D0 (Fig. 4G; YAP-DKD), while the expression of cleaved-caspase 3 in WT did not show a sharp contrast compared to its control D0. These results confirm the higher level of apoptosis in differentiated YAP-DKD cells during the later stages of spermatogenesis.

### Modulation of YAP expression at specific time points during spermatogenic differentiation of wild-type hESCs recapitulates the phenotypes of the YAP-KD cells

To confirm that an initial depletion followed by an up-regulation of YAP promotes spermatogenic differentiation of hESCs, we modulated the expression level of YAP at specific time points during spermatogenic differentiation of wild-type hESCs using two small molecules. Dobutamine hydrochloride (DH), which has previously been shown to promote YAP phosphorylation and suppress YAP activity^38,39^, was used to inhibit YAP expression, while lysophosphatidic acid (LPA) activated YAP expression in wild-type hESCs. DH-treated hESCs showed a 50% reduction in YAP level compared to untreated hESCs (Supplementary Fig. S7B,C), and also expressed lower levels of YAP target genes, *CTGF* and *CCND1,* compared to untreated hESCs (Supplementary Fig. S7D). Our results demonstrate that DH treatment successfully inhibits the expression of YAP and its target genes in wild-type hESCs. Although LPA-treated hESCs increased *CTGF*, *CCND1,* and *CYR61* expression levels compared to untreated hESCs (Supplementary Fig. S7D)*,* their YAP protein level did not increase compared to the untreated cells (Supplementary Fig. S7B,C). These results suggest that although LPA did not increase YAP level in hESCs, it might enhance YAP activity resulting in higher levels of YAP target genes.

**Figure 5:**
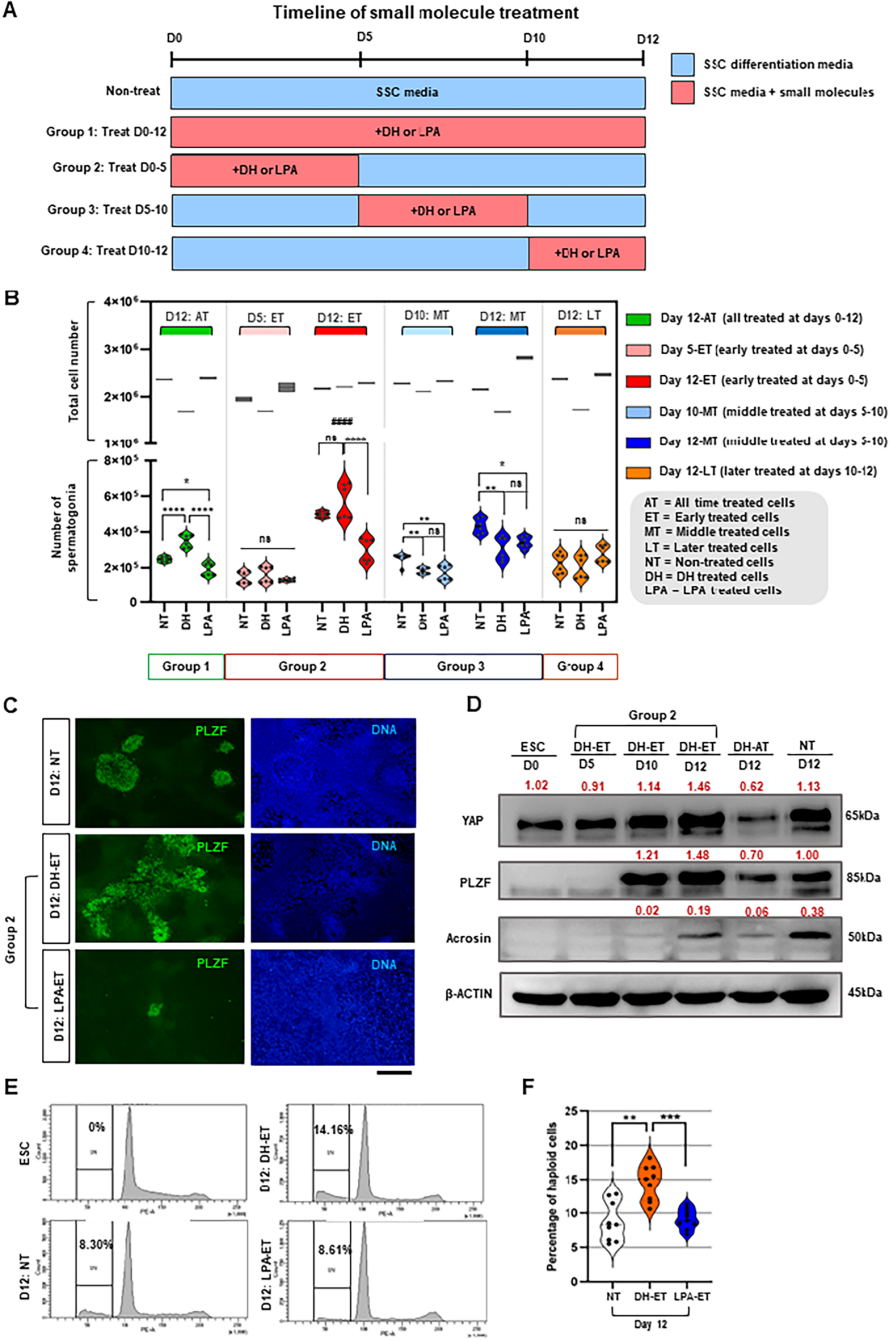
Modulation of YAP expression during the various stages of spermatogenic cell differentiation of wild-type hESCs by using DH and LPA. (**A**) Diagrams show the timeline of DH and LPA supplementation during spermatogenic differentiation of wild-type hESCs. (**B**) Graphs show the total number of differentiated hESCs and the number of PLZF^+^ hSSCs derived from various conditions described in (**A**) at the end of the culture. Data are presented as mean ± SD of 3 experiments. **P < 0.01, ***P < 0.001, ****P < 0.0001 and ###* P* < 0.001 indicated the statistically significant of spermatogonia number from DH treated cells between each group. (**C**) Representative immunofluorescence micrographs show the level of PLZF expression in DH-ET and LPA-ET cells compared to that of their untreated counterpart. Scale bar: 200 μm. (**D**) Western blot shows the expression of YAP, PLZF, Acrosin, and β-ACTIN proteins in DH-ET cells during their spermatogenic differentiation compared to those of DH-AT cells, untreated control cells, and non-differentiated hESCs. Red numbers indicate the expression level of each sample relative to β-ACTIN. (**E**) Analysis of DNA content by flow cytometry shows the percentage of haploid spermatid-like cells derived from DH-ET cells at the end of their spermatogenic differentiation compared to that of LPA-ET and untreated cells. (**F**) The graph shows the percentage of haploid spermatid-like cells derived from DH-ET cells at the end of their spermatogenic differentiation compared to that of LPA-ET and untreated cells. Data are presented as mean ± SD of 3 experiments. **P < 0.01, ***P < 0.001. The uncropped membranes associated with Fig. 5D are provided in Supplementary Fig. S11.

We then supplemented DH and LPA at various time points during spermatogenic differentiation of wild-type hESCs (Fig. 5A). Consistent with the results generated from YAP-KD cells, the highest number of hESC-derived hSSLCs was observed under the condition that DH was added to inhibit YAP expression during the first 5 days of spermatogenic differentiation before being removed for the rest of the culture period (Fig. 5B, D12: DH-ET in Group 2). On the contrary, hESCs treated with LPA to increase YAP activity during the first 5 days of their spermatogenic differentiation generated a lower number of hSSLCs than both D12: DH-ET and untreated cells (Fig. 5B, D12: LPA-ET in Group 2).

Unlike YAP suppression during the early stage of spermatogenic differentiation (days 0–5), suppressing YAP during the middle phase (days 5–10) of spermatogenic differentiation reduced the number of hESC-derived hSSLCs compared to untreated hESCs (Fig. 5B, D12: DH-MT in Group 3). Moreover, suppressing YAP during the middle stage (DH-MT in Group 3) or throughout the spermatogenic differentiation (DH-AT in Group 1) also reduced the total number of differentiated hESCs in culture (Fig. 5B), which could result in the decrease in the number of hESC-derived hSSLCs at the end of culture. This is consistent with our previous result with YAP-DKD cells showing that continued suppression of YAP during the later stages of spermatogenic differentiation increased the apoptotic level of differentiated hESCs (Fig. 4F,G).

Furthermore, our immunofluorescence study shows that the level of PLZF expression in DH-ET cells on day 12 was higher than that of the untreated control, while the level of PLZF expression in LPA-ET cells was lower than that of the untreated control (Fig. 5C). Western blot analysis further confirmed that DH-ET cells expressed higher levels of PLZF than the untreated control at the end of the culture (Fig. 5D), while LPA-ET cells expressed lower levels of PLZF than the untreated control at the end of the culture (Supplementary Fig. S7E,F). Unlike suppression of YAP during the early stage of spermatogenic differentiation (DH-ET), suppression of YAP throughout spermatogenic differentiation (DH-AT) decreased the expression level of PLZF compared to the untreated control (Fig. 5D). Although DH-ET treatment produces a higher number of haploid germ cells than the control, the level of Acrosin expression in the DH-ET group is lower than that of the control (Fig. 5D). Because the level of Acrosin is associated with spermatid maturity, it is possible that while DH-ET treatment increases the number of hESC-derived spermatids, these spermatids may be less mature than those derived from the untreated control.

We further investigated haploid germ cell production using cell cycle analysis to observe DNA content of the cells. The DH-ET cells also generate significantly higher percentages of haploid spermatid-like cells at the end of culture than their untreated counterpart (14.16 ± 2.61% vs 8.30 ± 2.86%) (Fig. 5E,F). These results support our hypothesis that inhibition of YAP activity during the early stage of spermatogenic differentiation facilitates the derivation of hSSLCs and spermatid-like cells from hESCs. In contrast, inhibition of YAP activity during the later stages of spermatogenic differentiation reduced the number of hESC-derived hSSLCs and spermatid-like cells, possibly by increasing the apoptotic rate of hSSLCs and preventing their maturation into haploid spermatid-like cells.

## Discussion

This study demonstrates a novel role for the Hippo-YAP signaling pathway in the regulation of spermatogenic differentiation of hESCs. We found that dynamic expression of YAP is crucial for hESCs to differentiate toward hSSLCs and spermatid-like cells, and depleting YAP during the early stage of spermatogenic differentiation improves the derivation of spermatogenic cells from hESCs.

In this study, we successfully derived haploid spermatid-like cells from hESCs using a spermatogenic differentiation culture. Our result shows that the hESC-based model could be applied to study the development of human spermatogenesis^25,28–31,40^ without the need to use an invasive procedure to obtain testicular tissues from patients. We also show that inhibition of YAP activity during the early stage of spermatogenic differentiation facilitates the derivation of hSSLCs from hESCs. This result agrees with a previous study showing that YAP depletion in mouse SSCs (mSSCs) up-regulates the expression levels of the spermatogenesis and oogenesis-specific basic helix loop helix 1 (*Sohlh1*) and neurogenin 3 (*Ngn3*) genes that are essential for their differentiation^11,41^. A recent study also found that the inactivation of YAP/TAZ increased the levels of follicle-stimulating hormone (FSH), luteinizing hormone (LH), and testosterone in adult male mice^14^, which could, in turn, act on testicular somatic cells (Sertoli cells and Leydig cells) to support spermatogenesis and maturation of spermatogenic cells. As there is an increased level of apoptosis in YAP-DKD cells, it is possible that the decreased survival of hESCs in spermatogenic differentiation media might indirectly affect their differentiation. This agrees with a previous showing that a deficiency of TAZ, a YAP homolog, increases the level of apoptosis and senescence in spermatogenic cells and Leydig cells after aging^15^. This result supports our hypothesis that hippo transcriptional mediators, such as YAP/TAZ, play a critical role in the survival and differentiation of male germ cells.

However, another previous study showed that YAP is dispensable for mouse spermatogenesis in vivo*,* since knocking out *YAP* in testicular germ cells (Yap^flox/flox^; Ddx4^cre/+^) did not affect sperm production in male mice^11^. This discrepancy could be caused by differences in microenvironmental factors between their in vivo and our in vitro systems. Some testicular somatic cells could release paracrine signals to rescue the effect of YAP depletion by up-regulating the levels of other proteins, such as TAZ, that could compensate for the loss of YAP function^42^, resulting in normal spermatogenesis in YAP knockout mice. The distinct spermatogenesis process between humans and mice, including differences in male germ cell biology, as well as the dynamics of germ cell proliferation and differentiation^43,44^ could also contribute to the discrepancy. Human male germline stem cells have been reported to have the largest pool of 22% undifferentiated spermatogonia compared to 0.3% of mouse germ cells^43^. The significantly higher number of undifferentiated spermatogonia in humans could partially explain why the effect of YAP depletion was clearly observed during our in vitro human spermatogenesis. Furthermore, human spermatogenic cells also have a shorter clonal expansion stage with only one transit-amplifying division of differentiated spermatogonia into spermatocytes, while differentiated mouse spermatogonia generally undergo 12 transit-amplifying divisions, resulting in a greater number of sperm production^43^. The longer expansion time of mouse spermatogonia could provide an opportunity for various molecular signals to compensate for the loss of YAP during mouse spermatogenic differentiation, therefore, only a small effect of YAP inactivation in murine spermatogenesis could be observed. We also found that the expression of acrosin, a marker of haploid spermatid-like cells derived from hESCs, could not be maintained in long-term culture, as demonstrated by the reduced level of Acrosin in differentiated YAK-KD cells on day 12 of culture compared to day 10. This could be due to the lack of testosterone and other testicular somatic cell-derived factors that are essential for the growth and maturation of spermatids under our culture conditions^45^.

PLZF, also known as ZBTB16 or ZNF145, is a multifunctional transcription factor that plays a critical role in maintaining the balance of progenitor and differentiated cells in various biological processes^6^, including spermatogenesis^4,5,44,46^. Mice lacking PLZF (*Zfp145*^-/-^) showed impaired spermatogenic cell development, progressive loss of mSSCs, and a decrease in the number of mature sperms^5^. This result suggests that PLZF is critical for the specification and maintenance of mSSCs during mouse spermatogenesis. As shown in this study, the expression level of YAP is well correlated with the expression level of PLZF during human spermatogenic differentiation. To our knowledge, we also demonstrate for the first time that YAP depletion during the early stage of spermatogenic differentiation increased PLZF expression during the later stages of spermatogenic differentiation of hESCs, leading to an increase in the number of hSSLCs and haploid spermatid-like cells. On the contrary, continued suppression of YAP throughout the spermatogenic differentiation results in a dramatic reduction in PLZF level and a reduced number of hESC-derived hSSLCs and haploid spermatid-like cells. These results suggest that the Hippo-YAP signaling pathway might control the spermatogenic differentiation of hESCs by regulating their PLZF expression. However, additional future experiments are needed to confirm this hypothesis.

## Conclusions

In this study, we demonstrate a novel function of the Hippo-YAP signaling pathway in human spermatogenesis using an hESC-based model. We successfully derived hSSLCs and haploid spermatid-like cells from hESCs and showed that dynamic expression of YAP during spermatogenic differentiation is essential for the derivation and maturation of hSSLCs and haploid spermatids. Modulating the level of YAP expression during human spermatogenesis could improve the production yield of male germ cells derived from hESCs, which could provide a valuable therapeutic cell source for male infertility in the future.

### Supplementary Information

Supplementary Figures.

Supplementary Tables.

Supplementary Legends.

## Data Availability

The datasets used and/or analyzed during the current study are available from the corresponding author on reasonable request.
