# Supplementary material for: The dynamic expression of YAP is essential for the development of male germ cells derived from human embryonic stem cells
Source: Sci Rep. 2024 Jul 8;14:15732. doi: 10.1038/s41598-024-66852-x (PMC11231333; doi:10.1038/s41598-024-66852-x)

Supplemental_Figure_S1

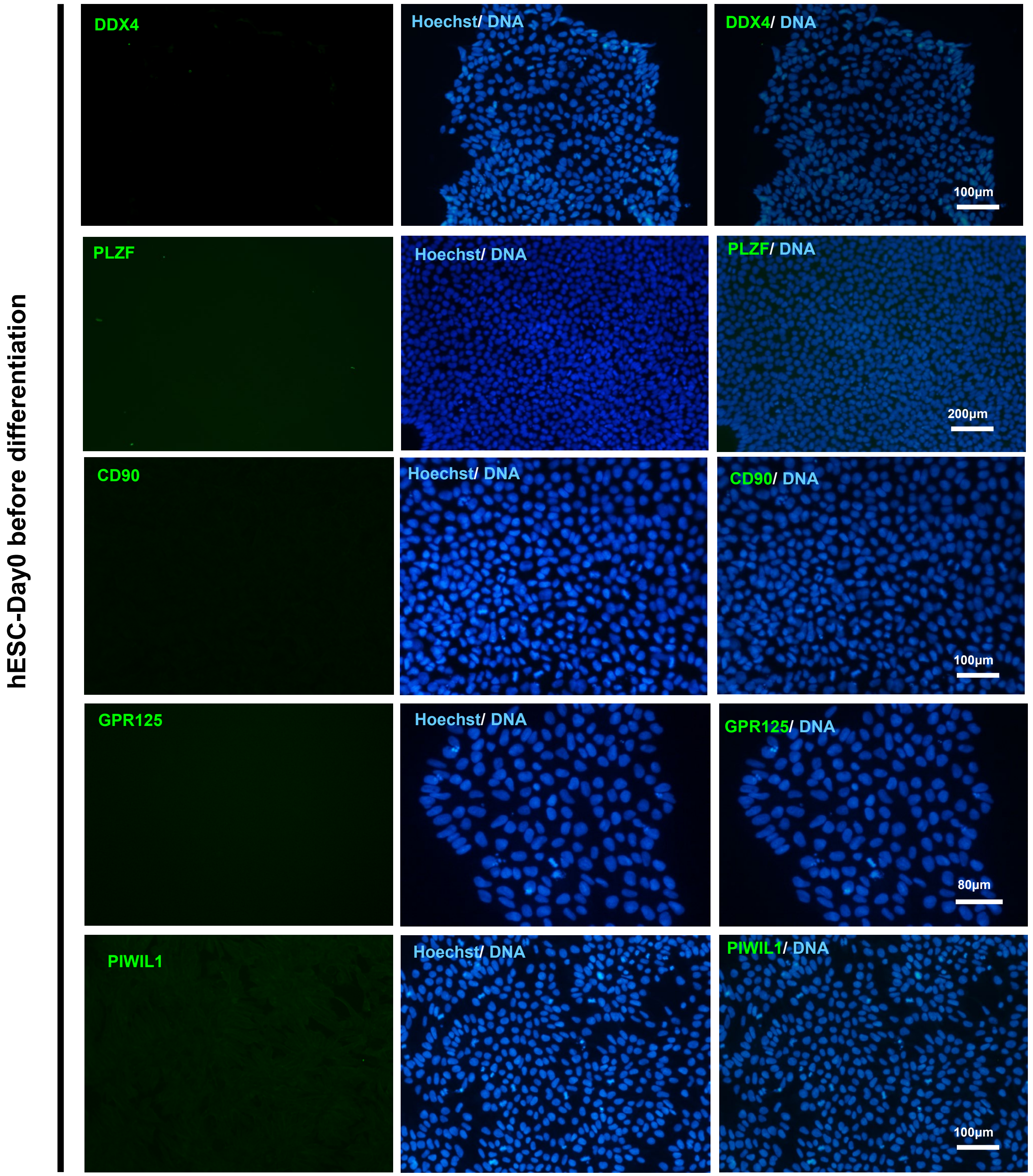

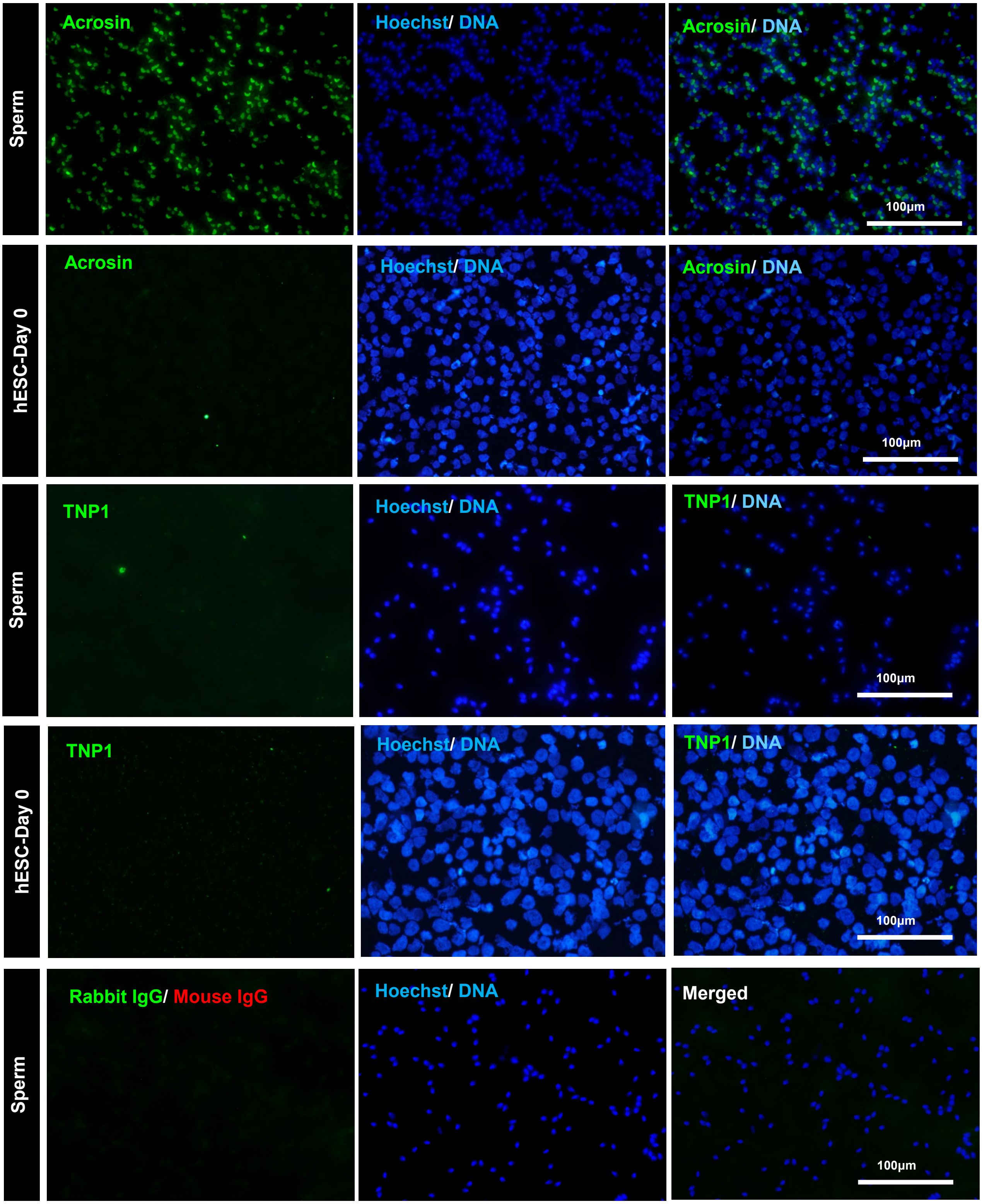
Sperm
Acrosin
Hoechst/ DNA
Acrosin/ DNA
100µm
hESC-Day 0
Acrosin
Hoechst/ DNA
Acrosin/ DNA
100µm
Sperm
TNP1
Hoechst/ DNA
TNP1/ DNA
100µm
hESC-Day 0
TNP1
Hoechst/ DNA
TNP1/ DNA
100µm
Sperm
Rabbit IgG/ Mouse IgG
Hoechst/ DNA
Merged
100µm

Supplemental_Figure_S3

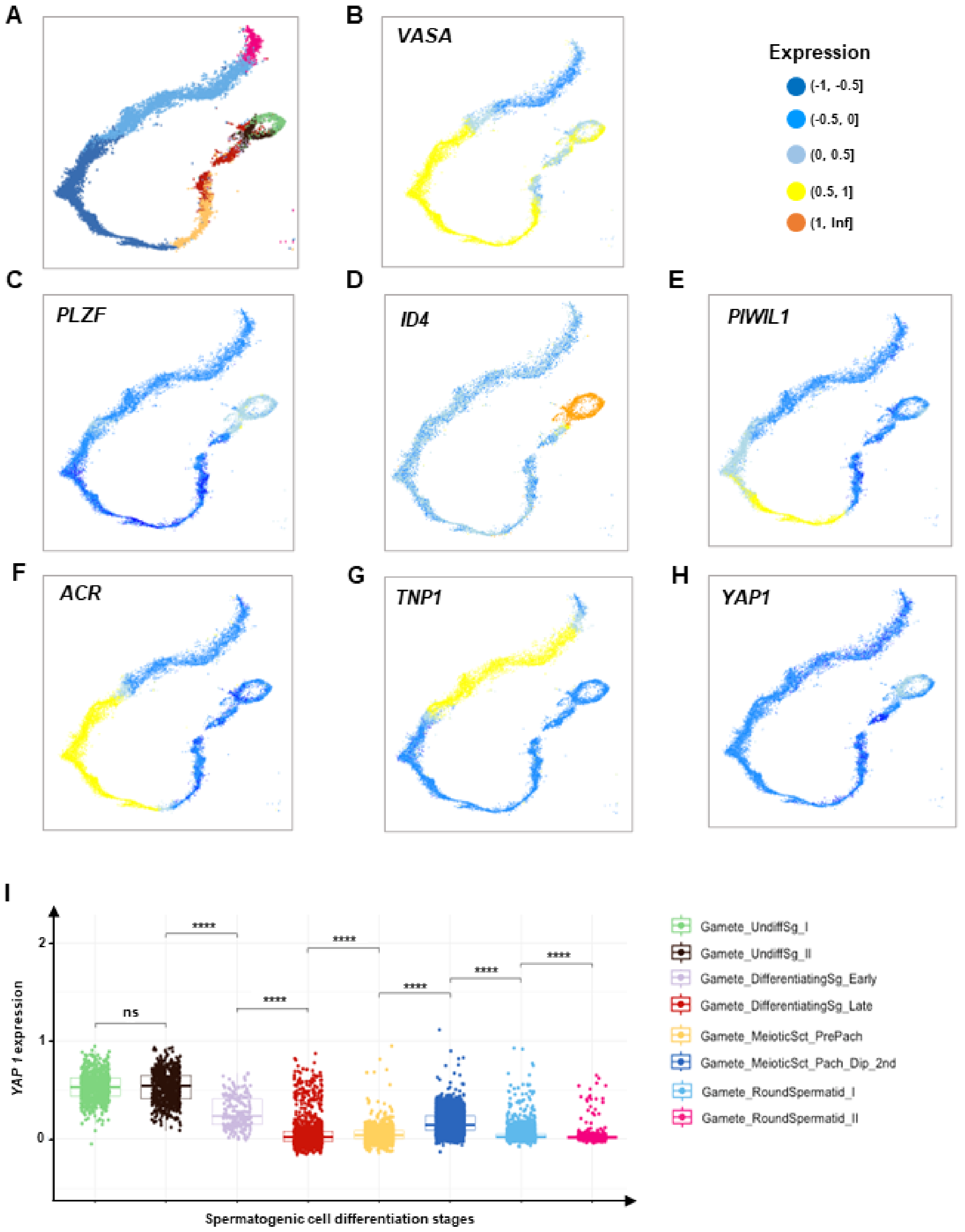

Supplemental_Figure_S4

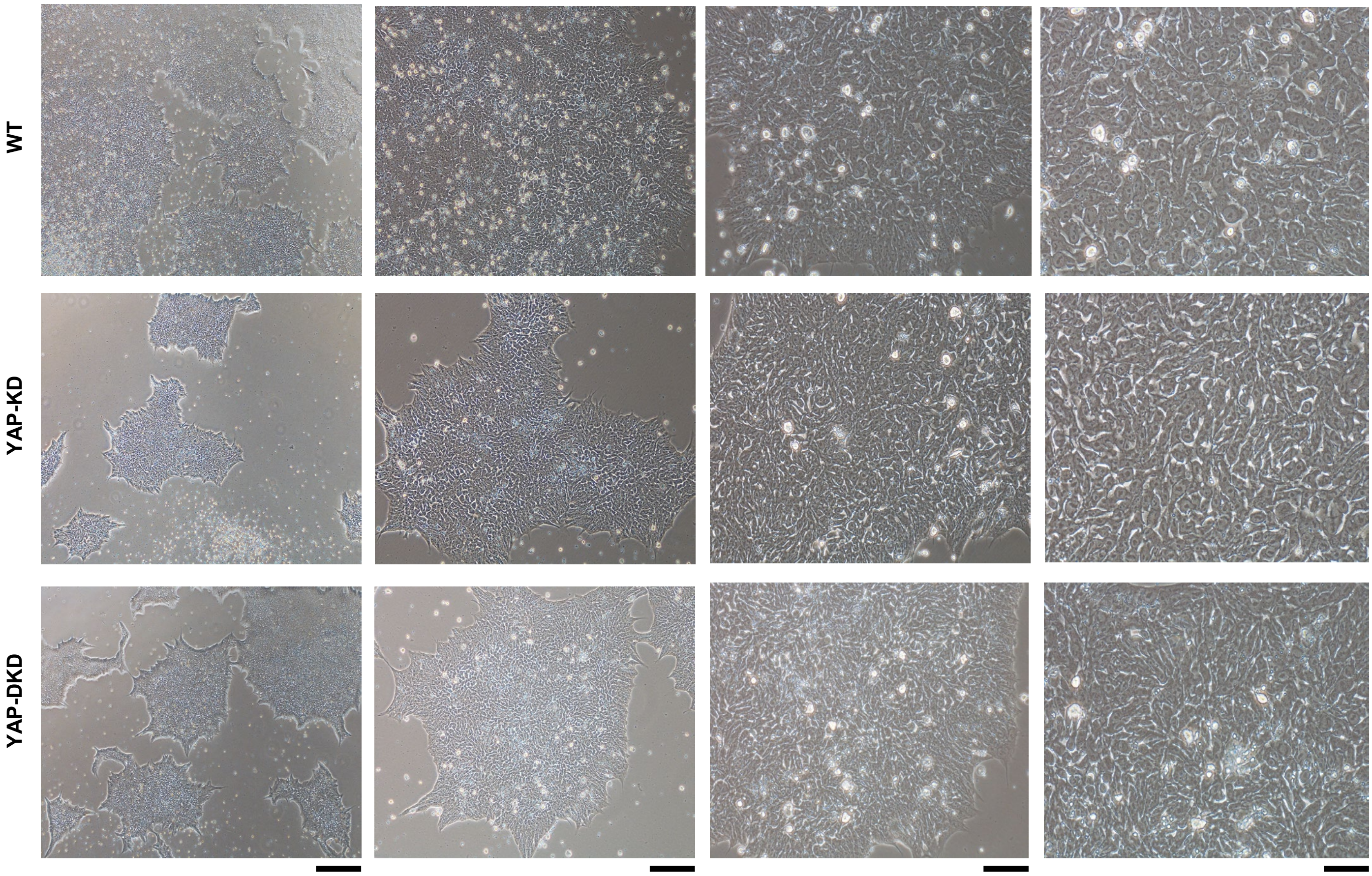

Supplemental_Figure_S5

WT: spermatogenic cell differentiation

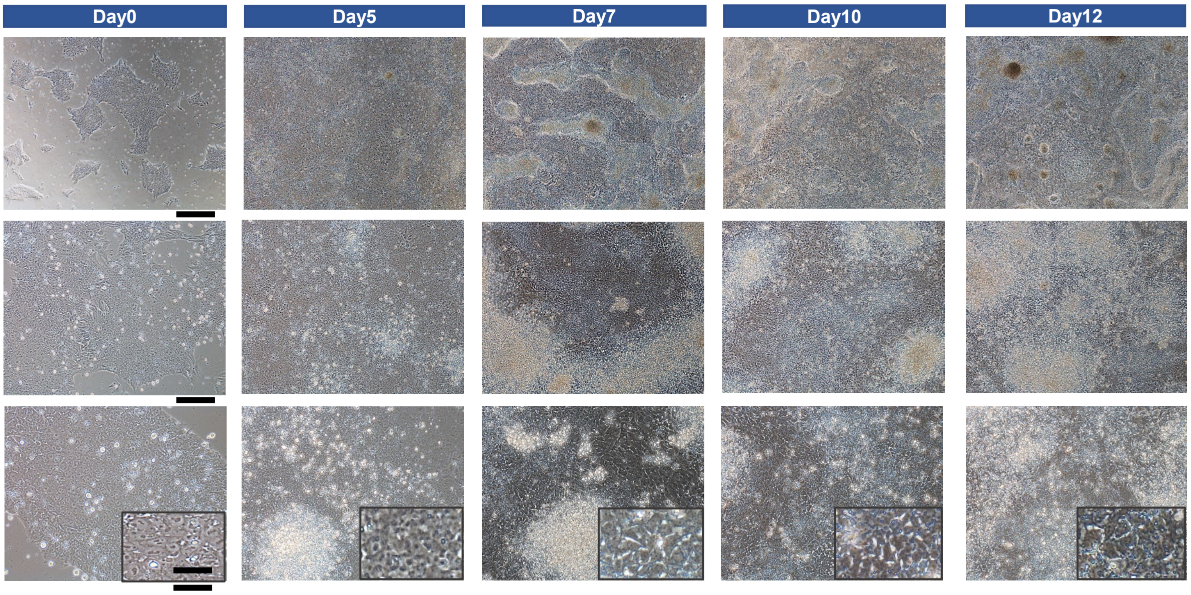

YAP-KD: spermatogenic cell differentiation

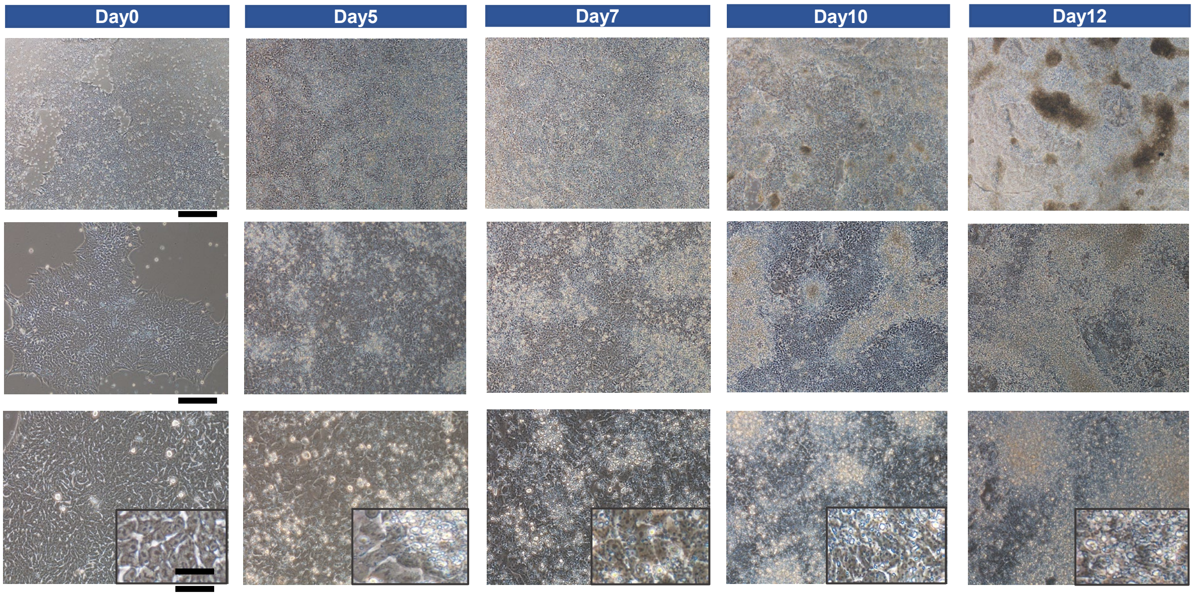

YAP-DKD: spermatogenic cell differentiation

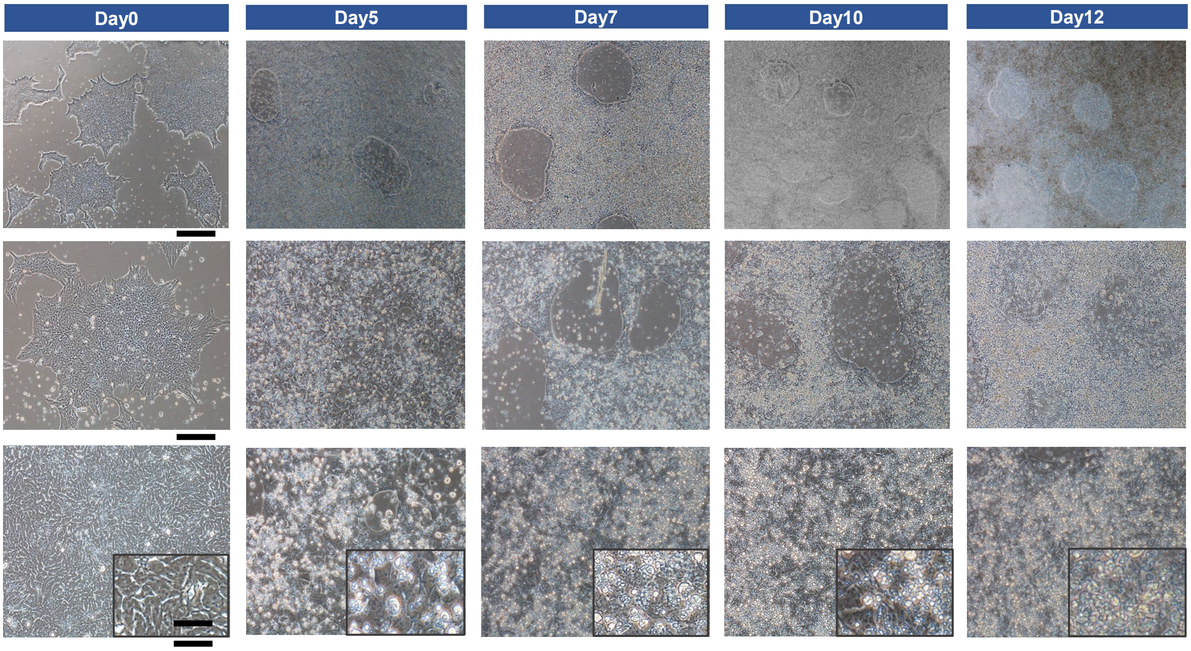

Supplemental_Figure_S6

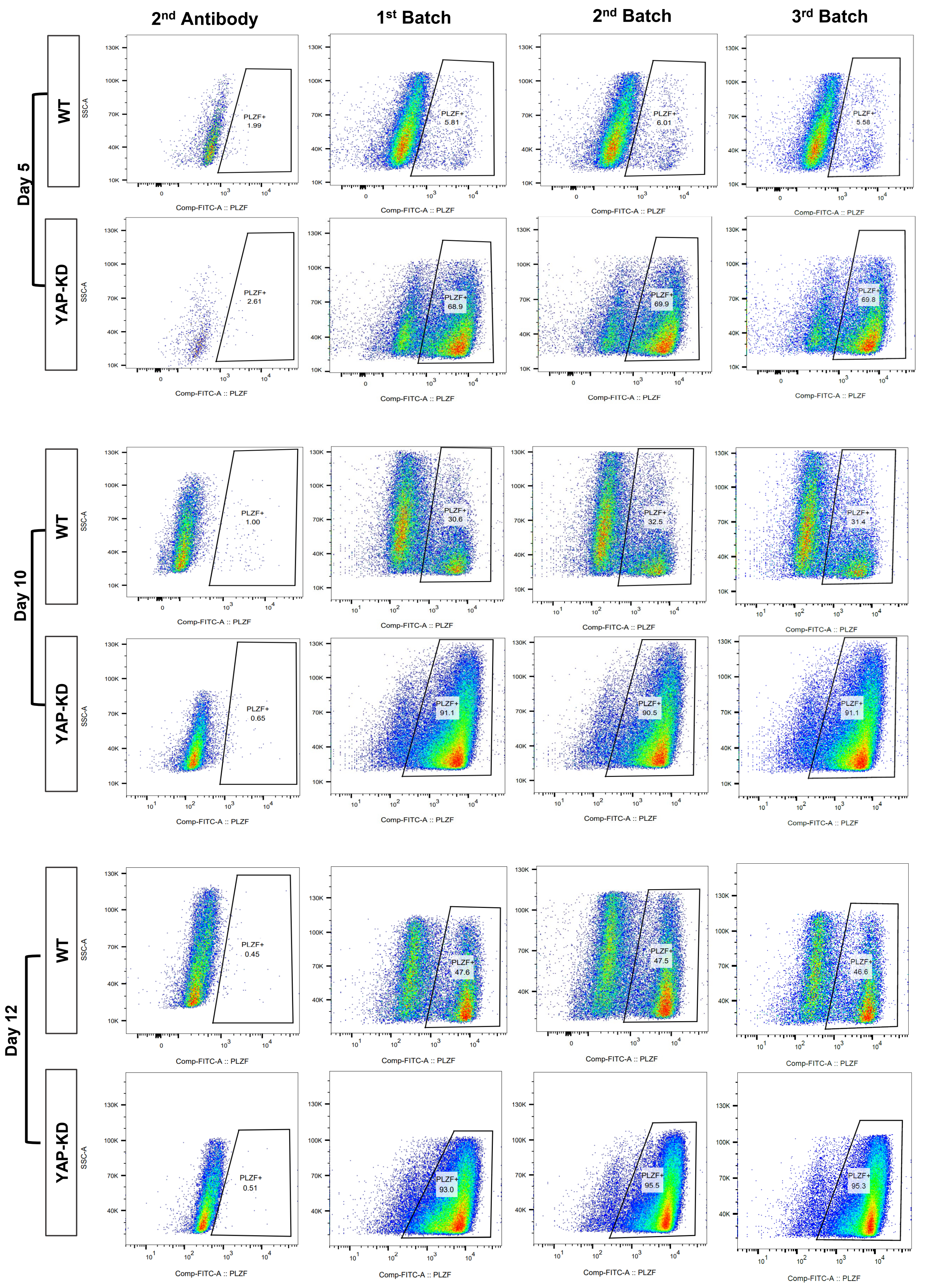

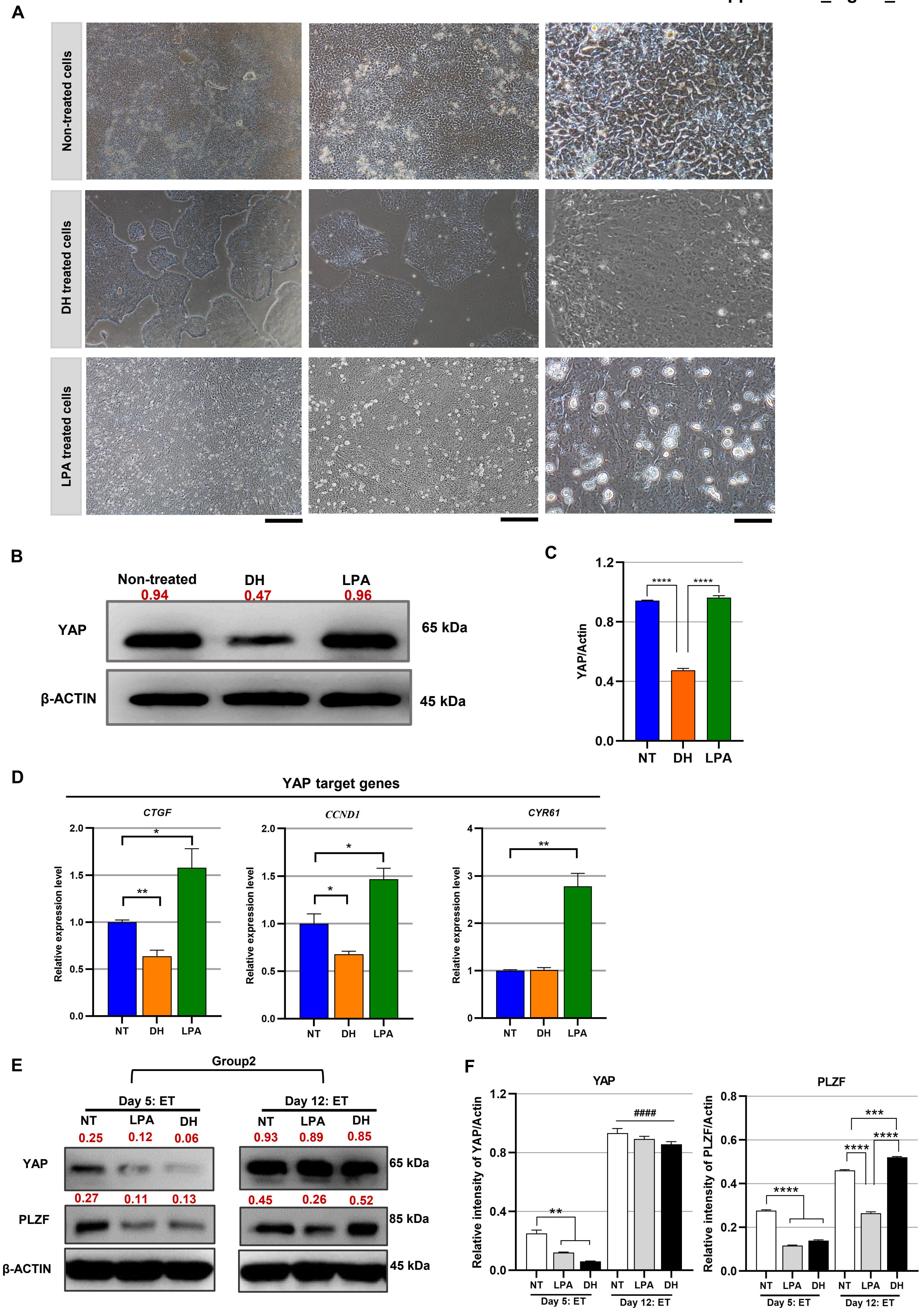
A
Non-treated cells
DH treated cells
LPA treated cells
B
Non-treated
DH
LPA
0.94
0.47
0.96
YAP
65 kDa
β-ACTIN
45 kDa
C
YAP/Actin
1.2
0.8
0.4
0.0
****
****
NT
DH
LPA
D
YAP target genes
CTGF
CCND1
CYR61
Relative expression level
2.0
1.5
1.0
0.5
0.0
4
3
2
1
0
*
**
NT
DH
LPA
E
Group2
Day 5: ET
Day 12: ET
NT
LPA
DH
0.25
0.12
0.06
0.93
0.89
0.85
YAP
65 kDa
0.27
0.11
0.13
0.45
0.26
0.52
PLZF
85 kDa
β-ACTIN
45 kDa
F
YAP
PLZF
Relative intensity of YAP/Actin
Relative intensity of PLZF/Actin
1.2
0.8
0.4
0.0
0.6
0.2
**
####
***
****
NT
LPA
DH
Day 5: ET
Day 12: ET

Supplemental_ Figure_S8 (Associated with figure 2)

Figure 2A

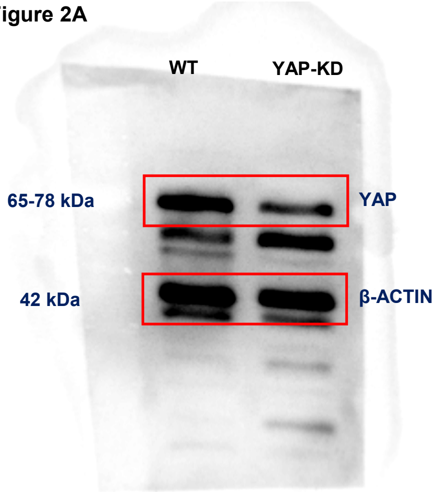

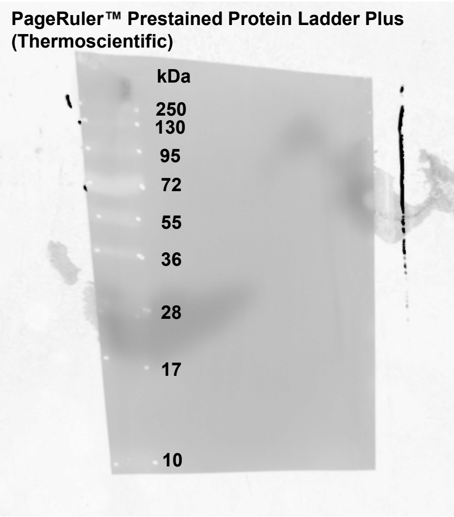

Figure 2G-1

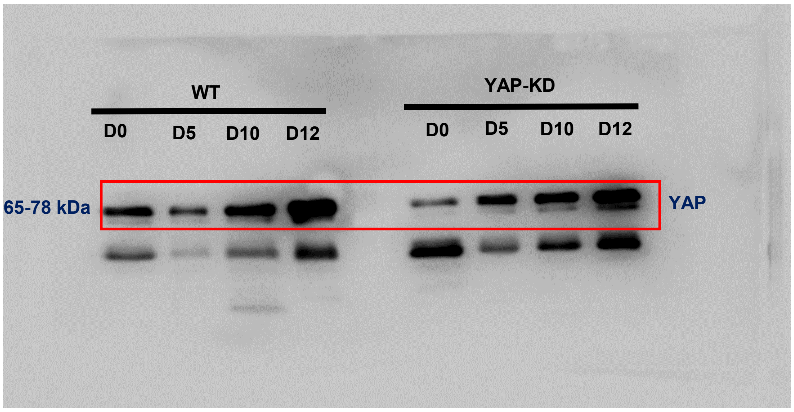

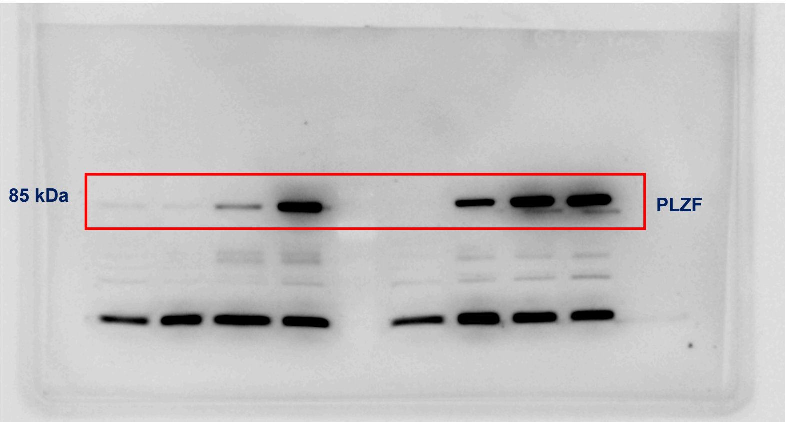

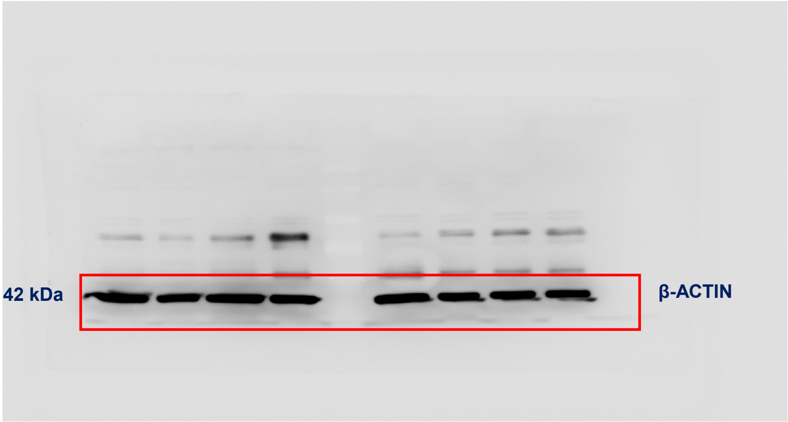

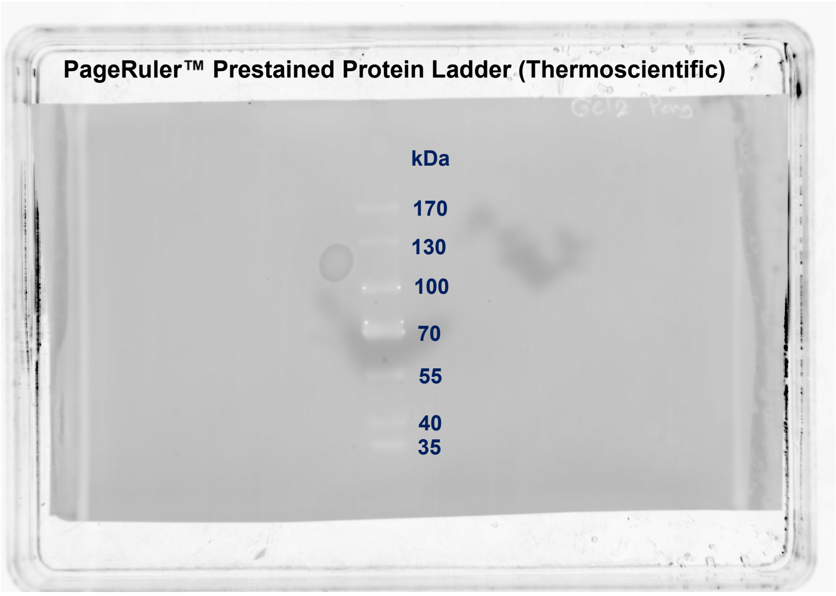

Figure 2G-2

Supplemental_ Figure_S8 (Associated with figure 2) continue

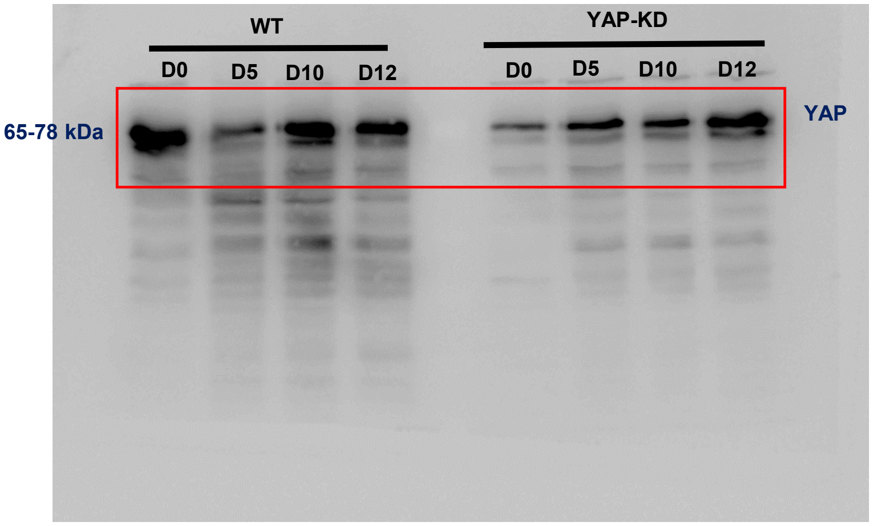

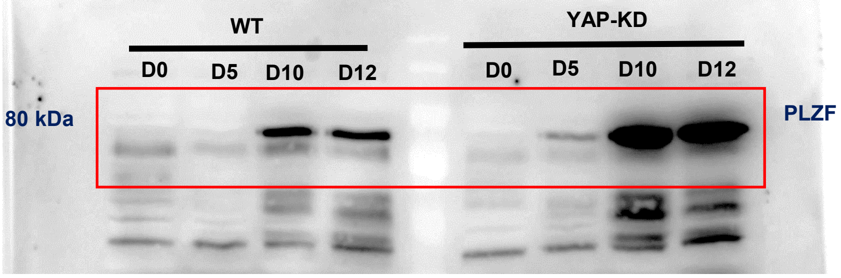

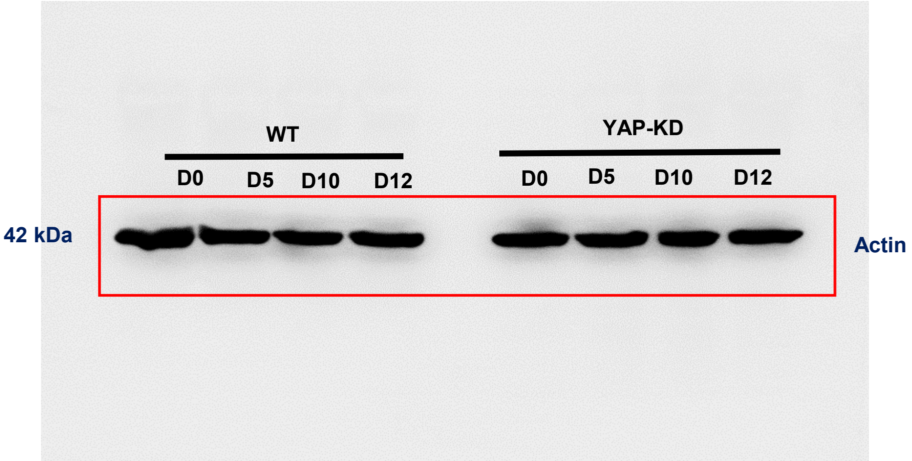

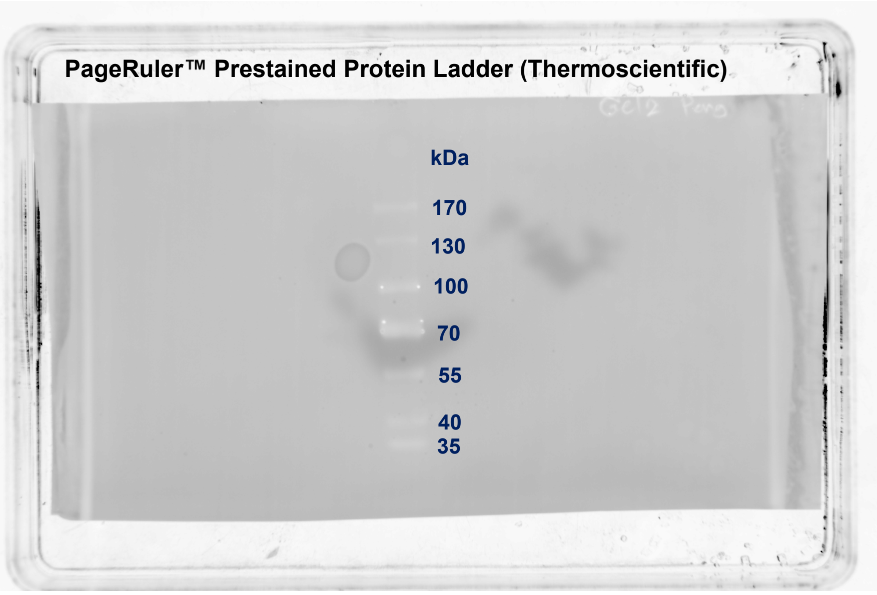

Supplemental_ Figure_S9 (Associated with figure 3)

Figure 3B

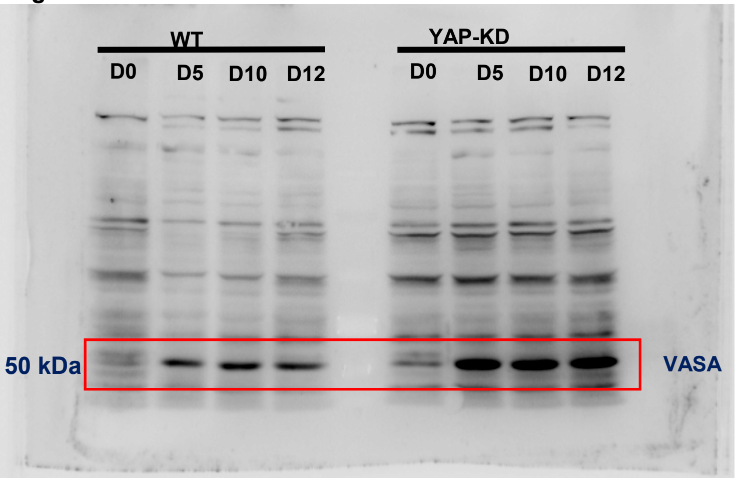

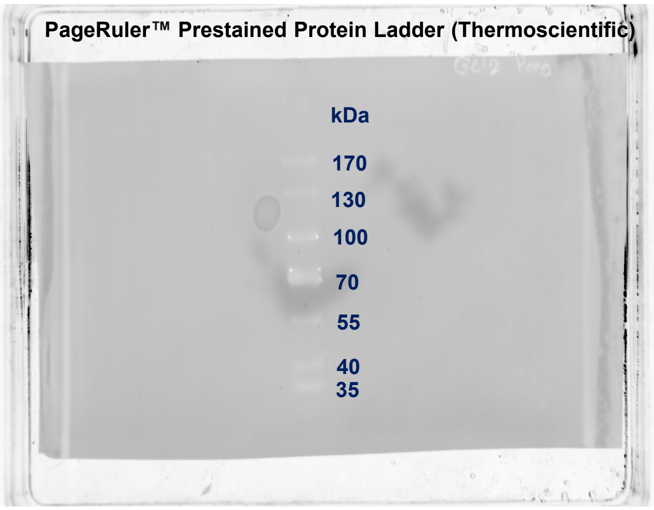

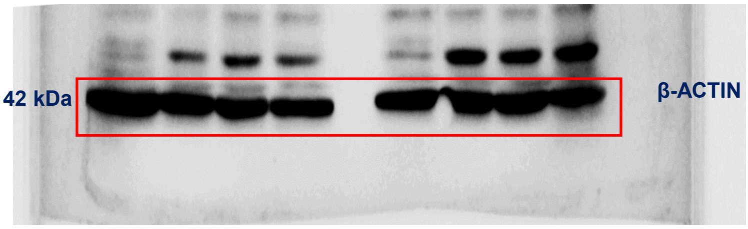

Figure 3D-1

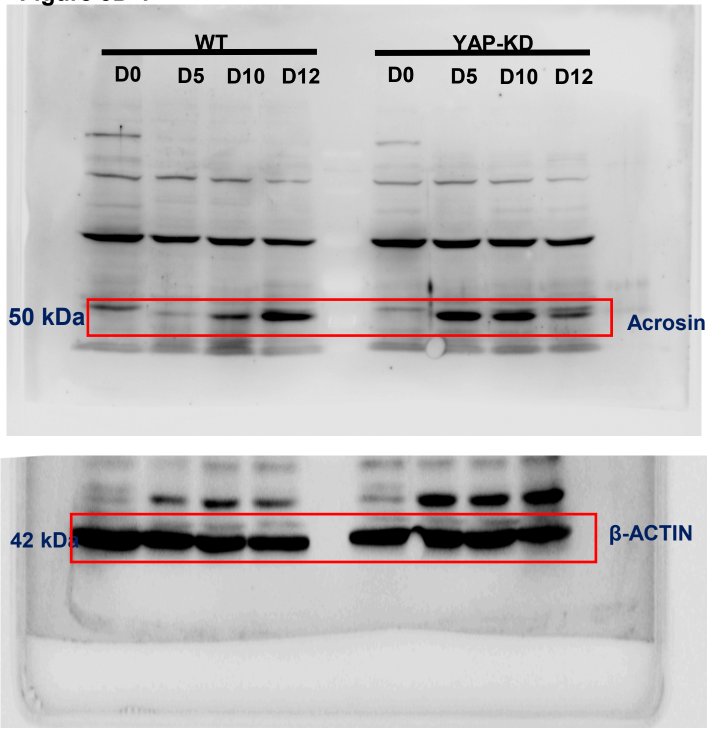

Figure 3D-2

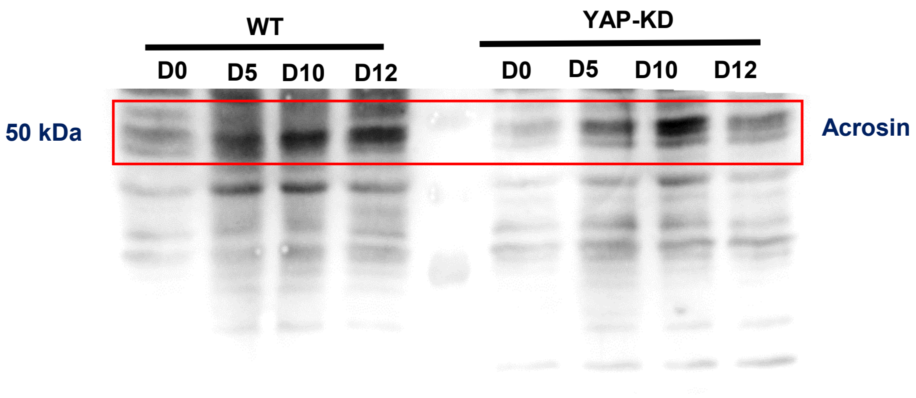
WT
YAP-KD
D0
D5
D10
D12
D0
D5
D10
D12
50 kDa
Acrosin

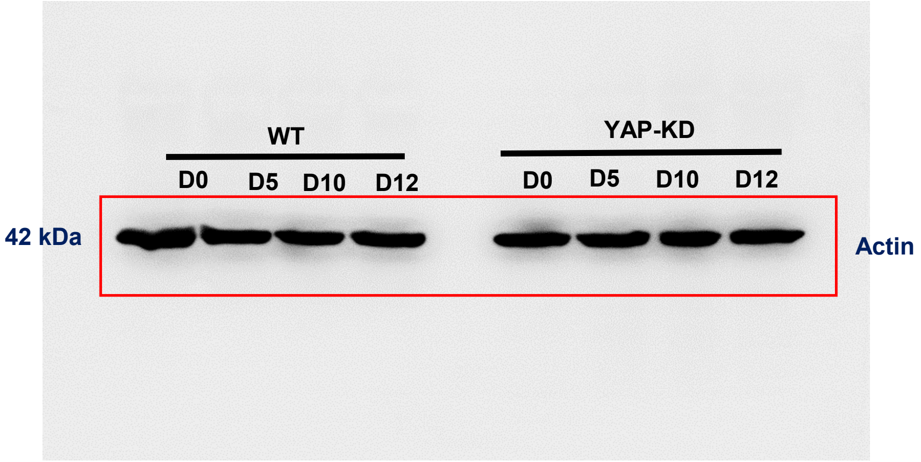
WT
YAP-KD
D0
D5
D10
D12
D0
D5
D10
D12
42 kDa
Actin

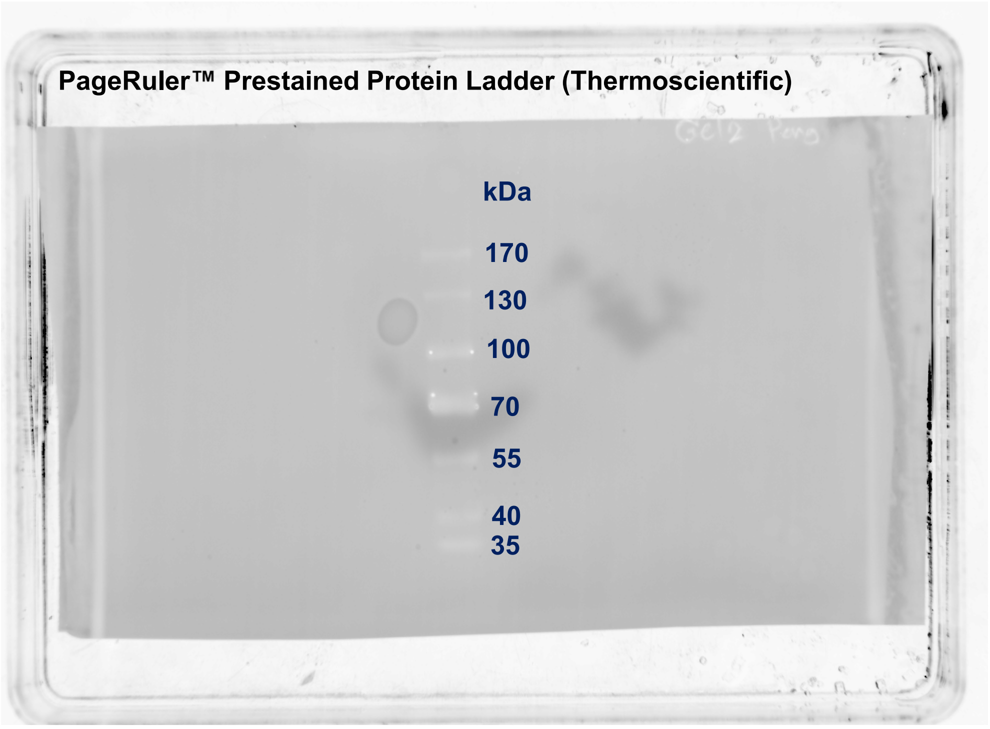
PageRuler™ Prestained Protein Ladder (Thermoscientific)
kDa
170
130
100
70
55
40
35

Supplemental_ Figure_S10 (Associated with figure 4)

Figure 4A

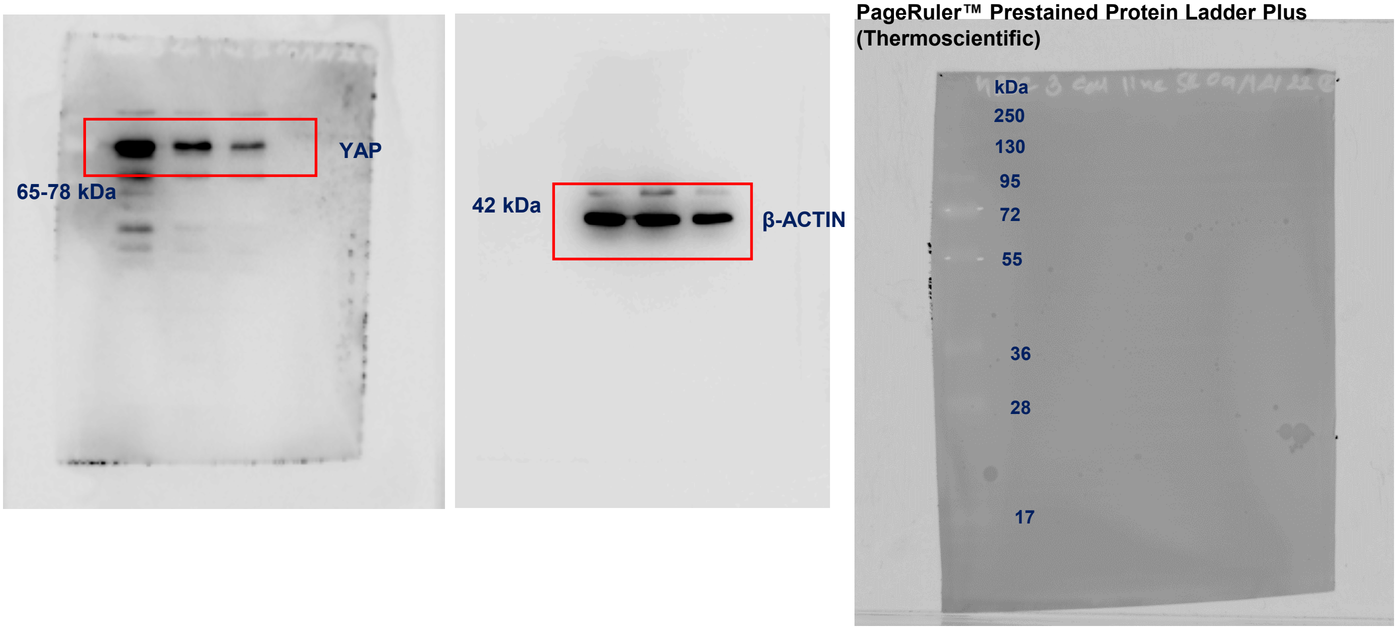

Figure 4C: WT

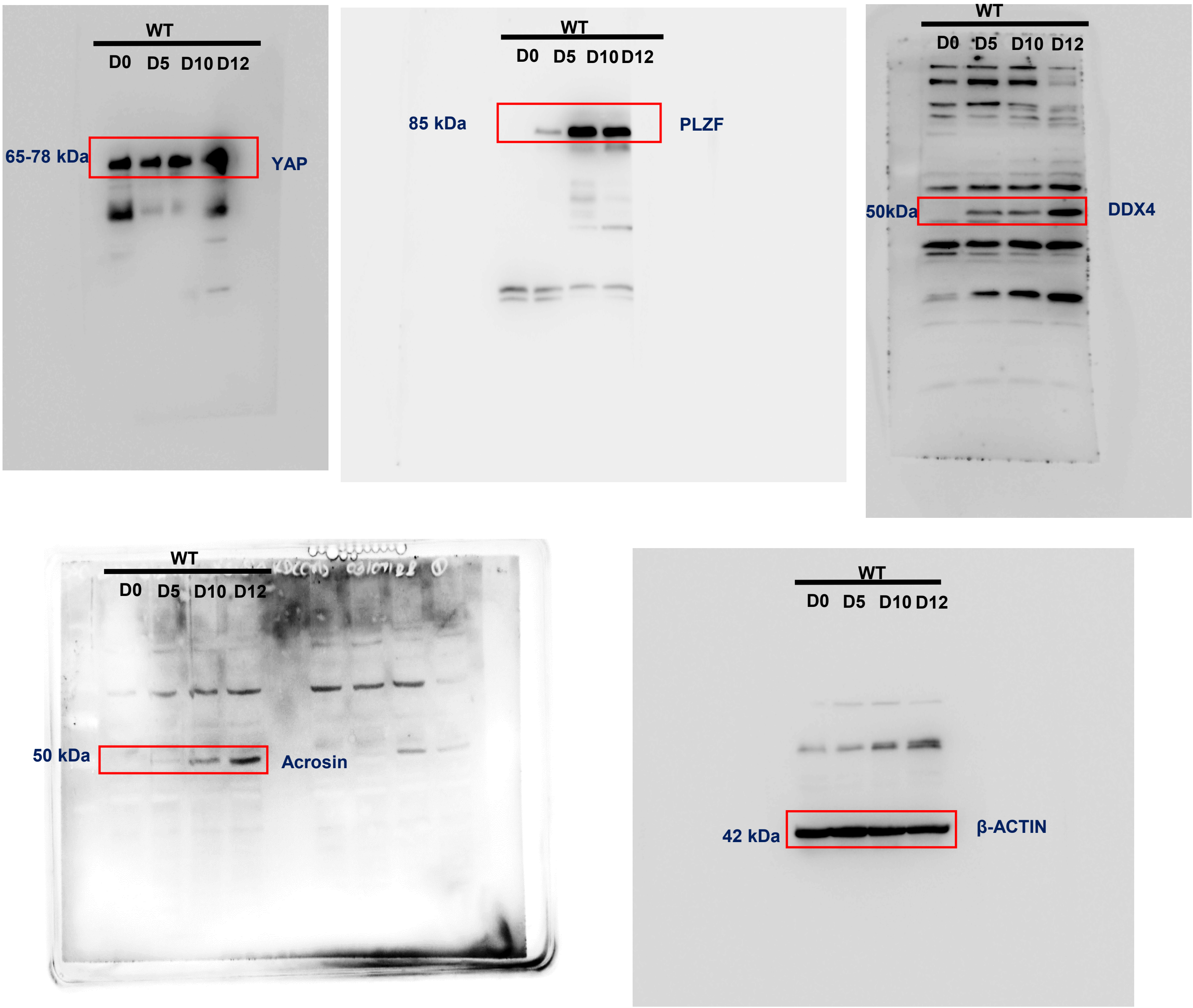

Supplemental_ Figure_S10 (Associated with figure 4) continue

Figure 4C: YAP-KD

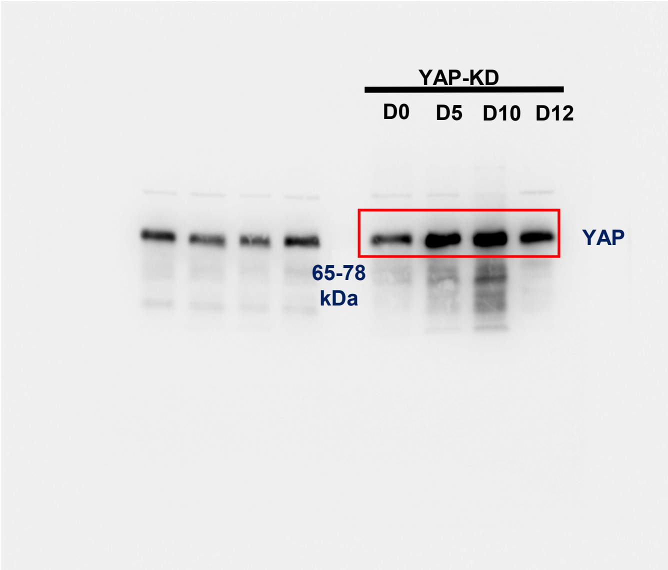

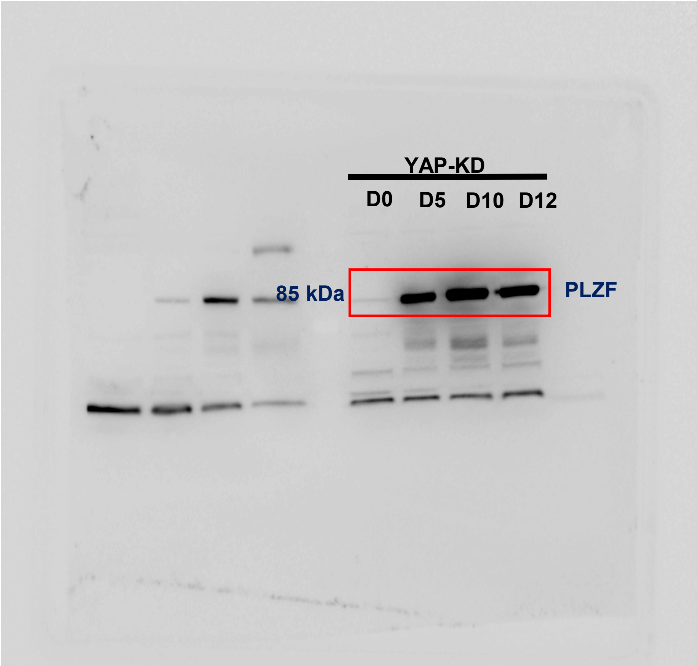

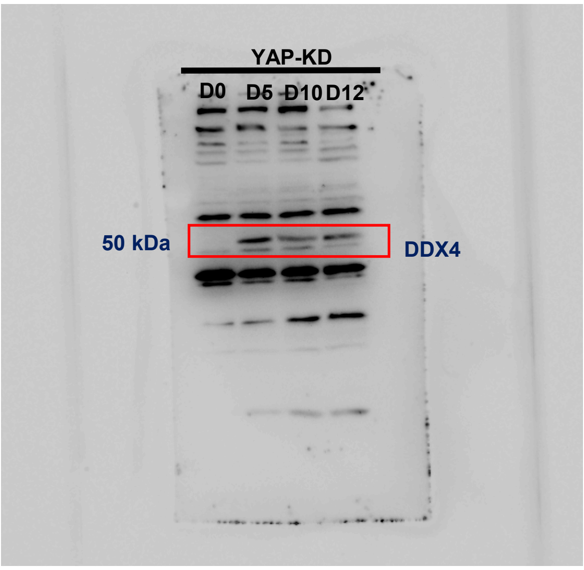

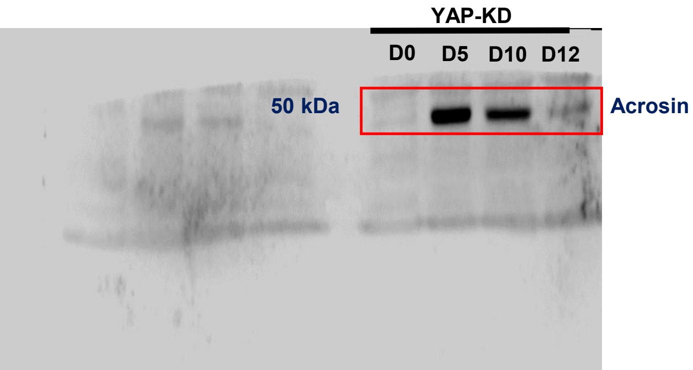

PageRuler™ Prestained Protein Ladder Plus (Thermoscientific)

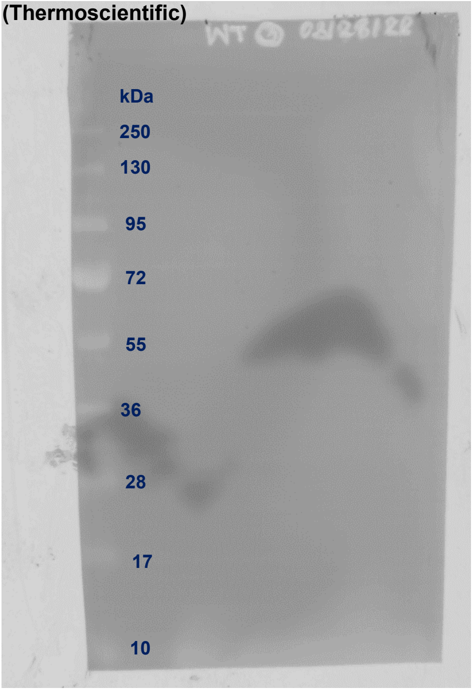

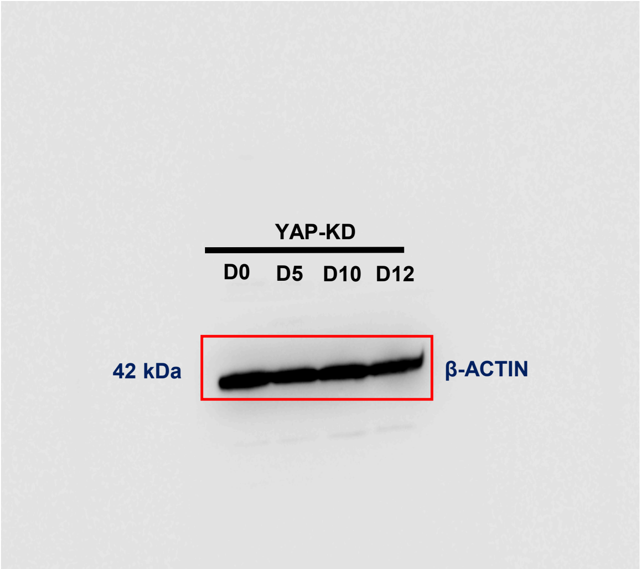

Figure 4C: YAP-DKD

Supplemental_ Figure_S10 (Associated with figure 4) Continue

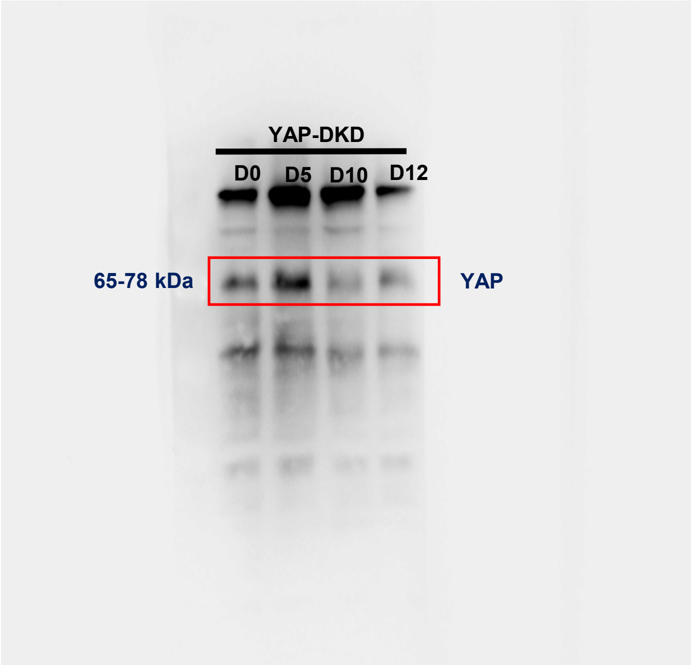

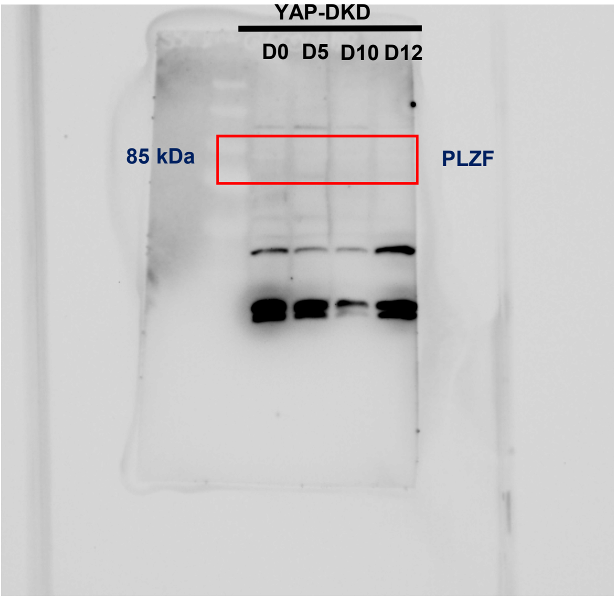

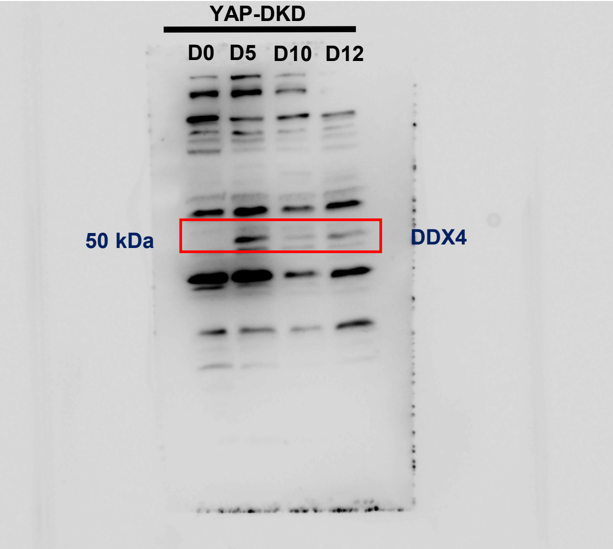

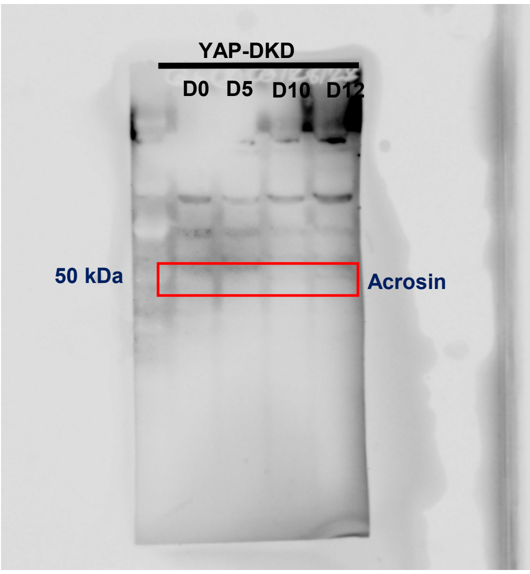

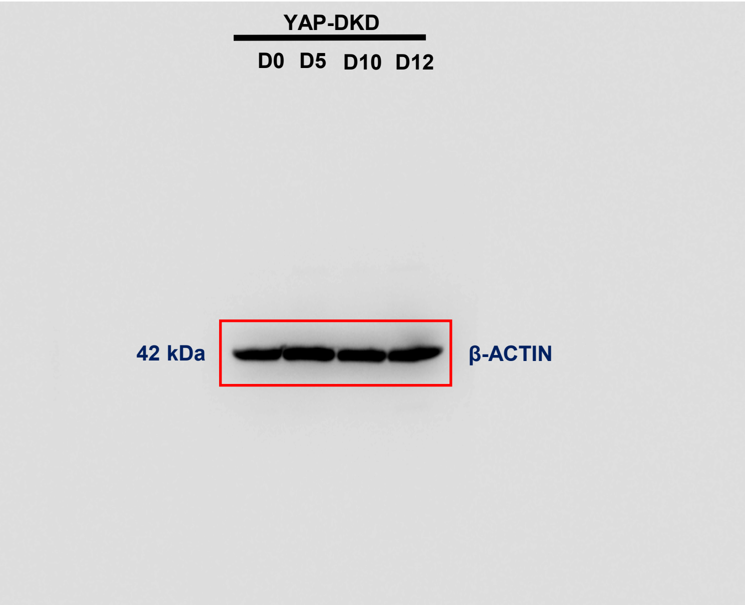

PageRuler™ Prestained Protein Ladder Plus (Thermoscientific)

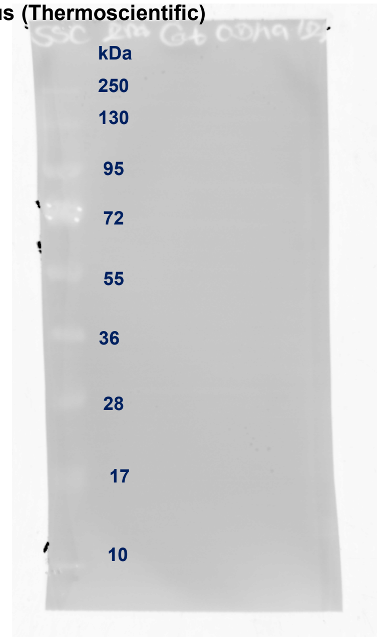

Supplemental_ Figure_S10 (Associated with figure 4) Continue

Figure 4G

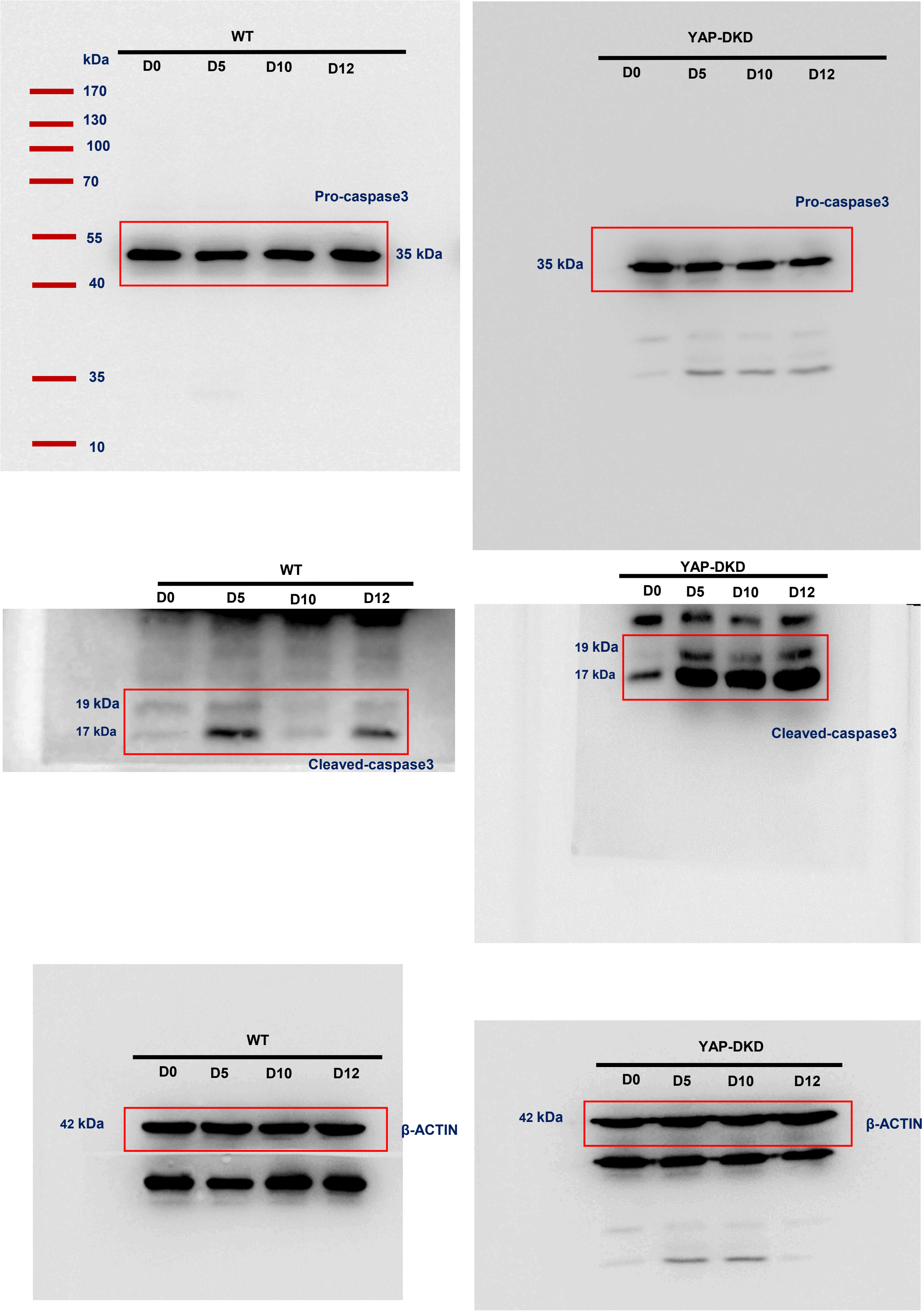

Figure 5D

Supplemental_ Figure_S11 (Associated with figure 5)

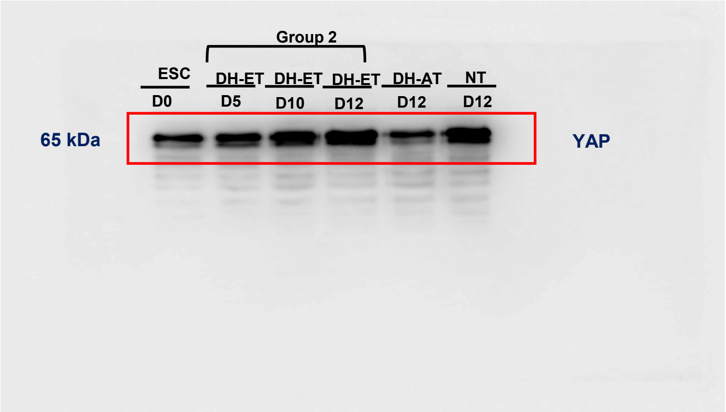

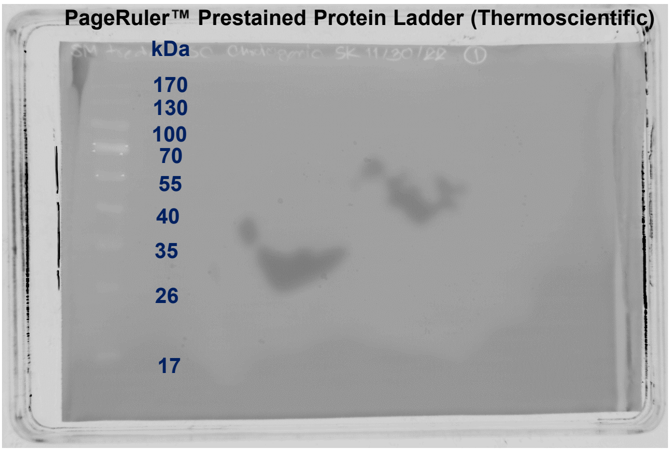

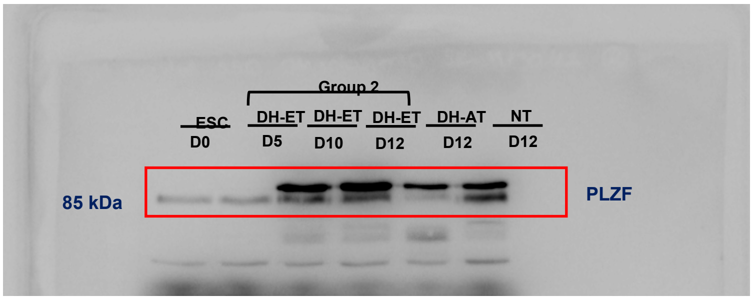

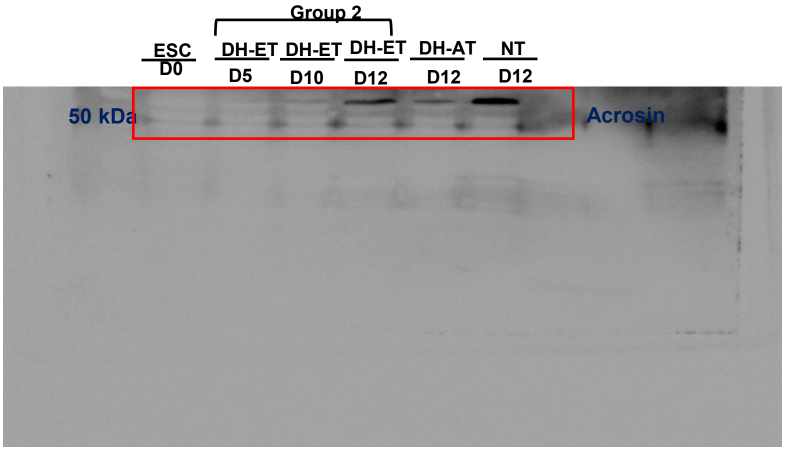

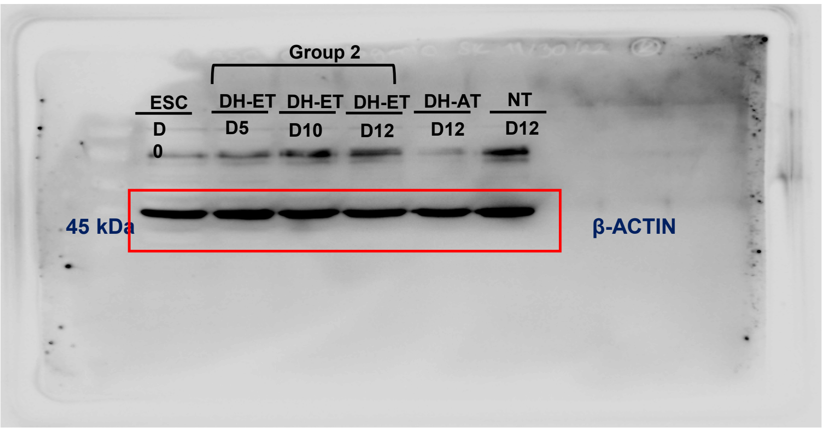

Supplemental_ Figure_S12 (Associated with Supplemental_figure_S7)

Supplemental_Figure_S7B

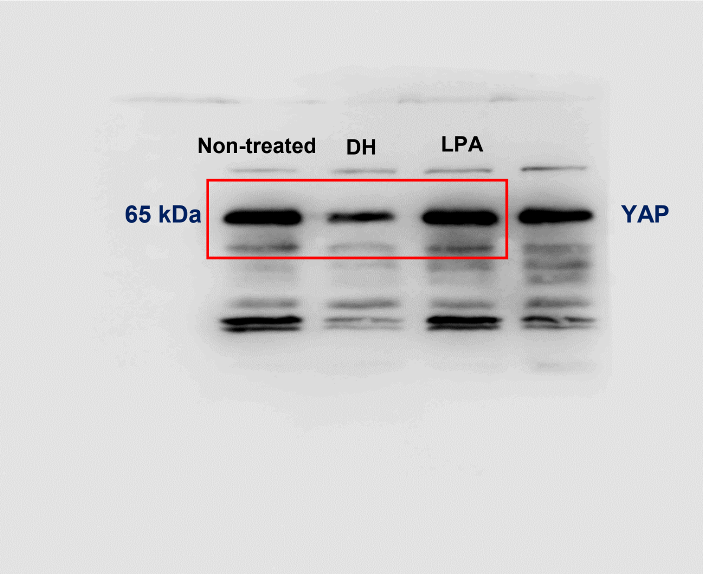

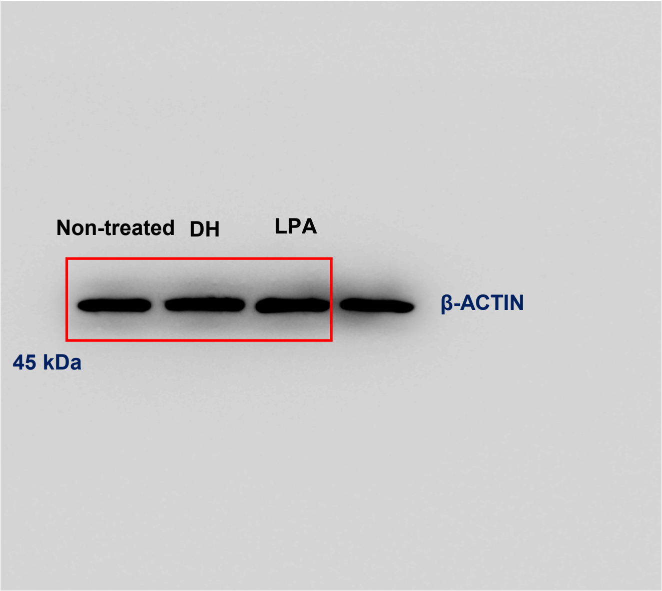

Supplemental_Figure_S7E

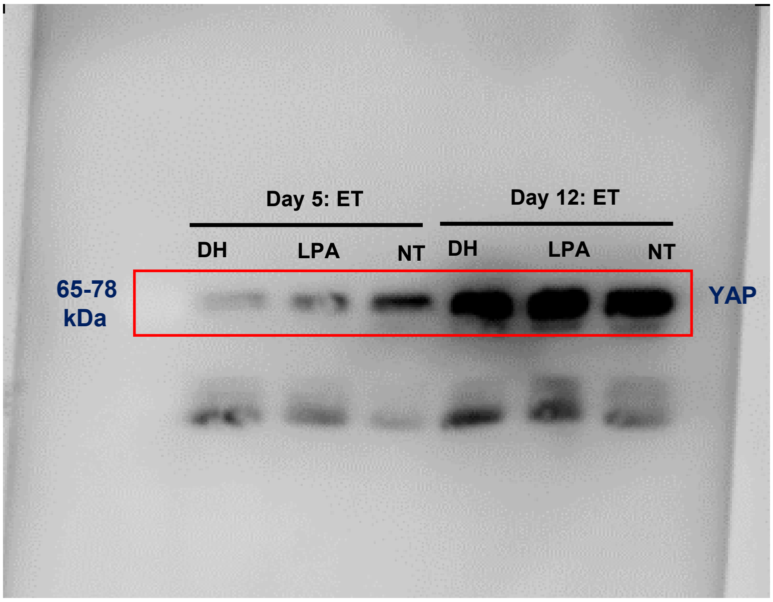

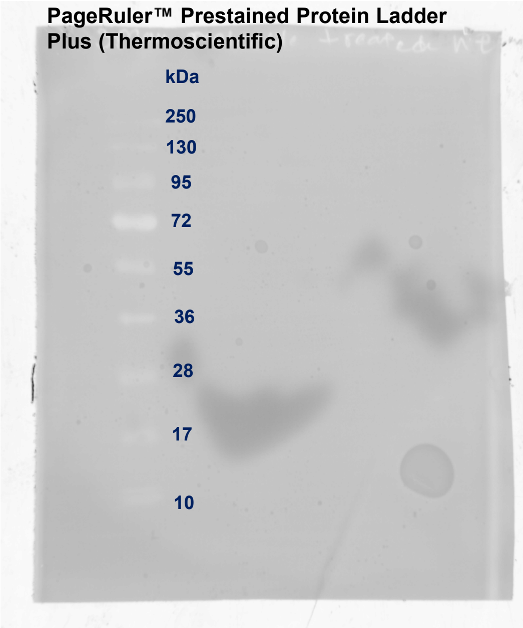

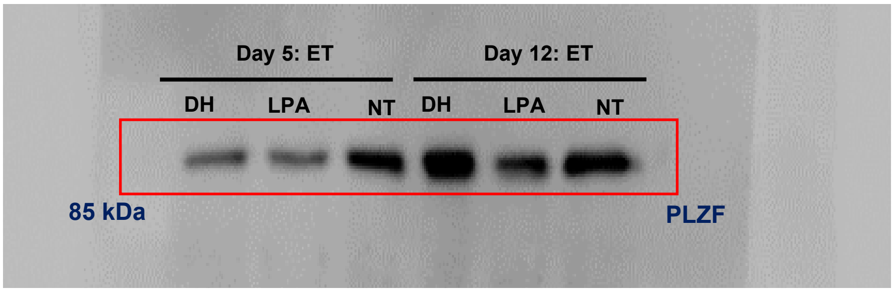

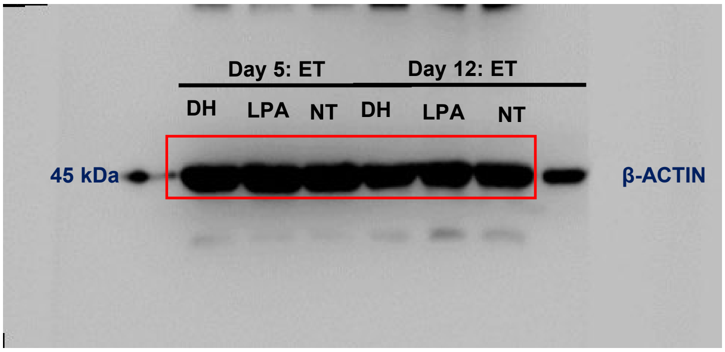

Supplemental_Figure_S13

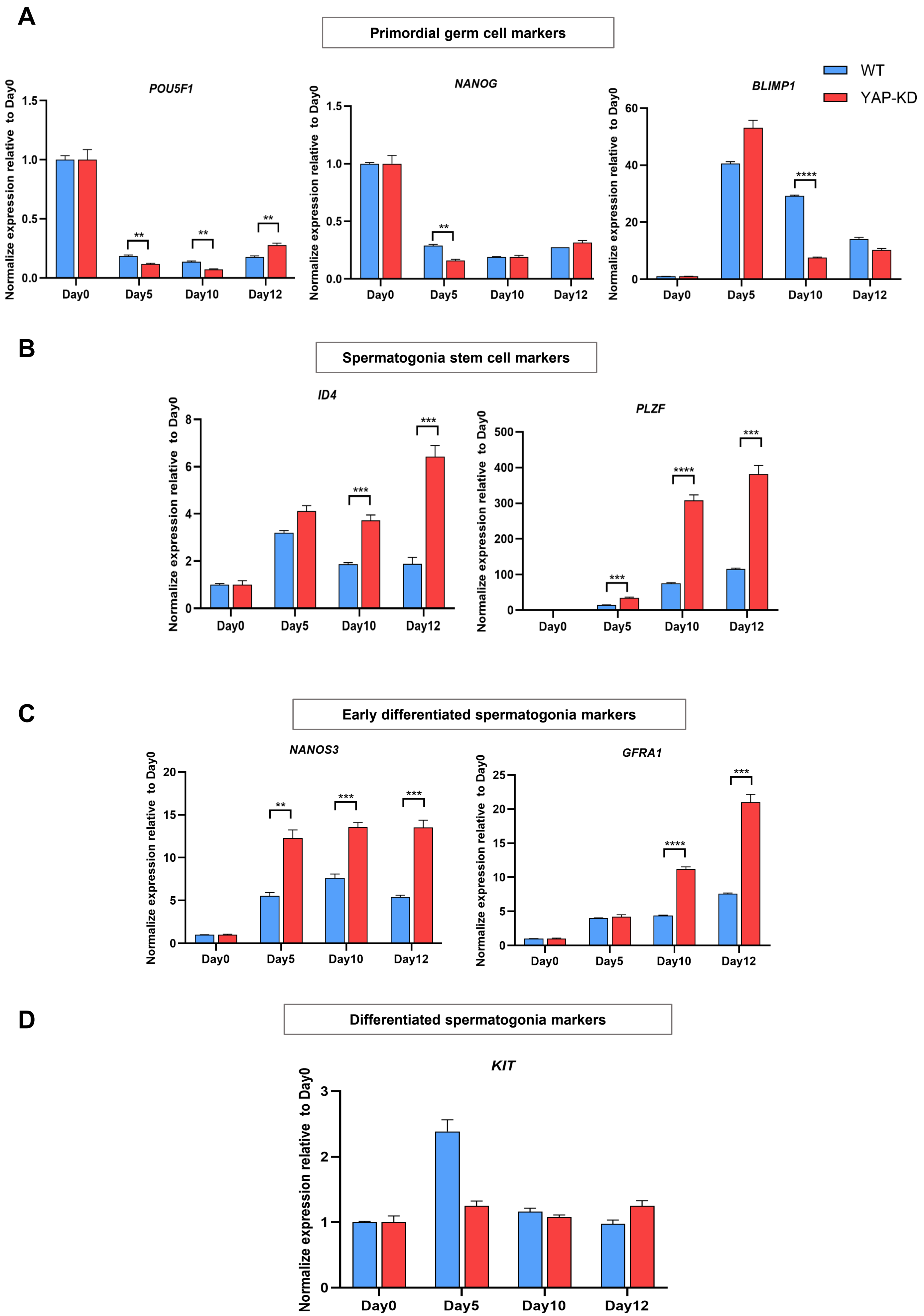

Supplement: Supplementary file 1 — Supplementary Figures. [file 41598_2024_66852_MOESM1_ESM.pdf]
