# Supplementary material for: The dynamic expression of YAP is essential for the development of male germ cells derived from human embryonic stem cells
Source: Sci Rep. 2024 Jul 8;14:15732. doi: 10.1038/s41598-024-66852-x (PMC11231333; doi:10.1038/s41598-024-66852-x)
Supplement: Supplementary file 2 — Supplementary Tables. [file 41598_2024_66852_MOESM2_ESM.docx]

**Supplementary table S1**: **List of primers and antibodies used in this study**

| **List of primers for qRT-PCR** | | |
| --- | --- | --- |
| **Gene name** | **Forward (5’🡪3’)** | **Reverse (5’🡪3’)** |
| *VASA* | CGTGTTTGCATCAGTTGATACC | CTGGATTGGGAGCTTGTGAA |
| *DAZL* | GCCACGTCCTTTGGTTTTTA | GACCTGAACTGGTGAATTTGG |
| *STELLA* | CATTCAAATGTCCCTGCAGTT | GCATAGAGTAGCTTTCTCAACCTG |
| *TFAP2C* | ATTAAGAGGATGCTGGGCTCTG | CACTGTACTGCACACTCACCTT |
| *BLIMP1* | TAAAGCAACCGAGCACTGAGA | ACGGTAGAGGTCCTTTCCTTTG |
| *SOX17* | TTCGTGTGCAAGCCTGAGAT | TAATATACCGCGGAGCTGGC |
| *PIWIL4* | ATGGCACCGAGATCACCTAT | GCTGAGCCTCACTGTTGTCA |
| *PLZF* | GAGATCCTCTTCCACCGCAAT | CCGCATACAGCAGGTCATC |
| *GFRA1* | CCAAGCACAGCTACGGAATG | CAGGCACGATGGTCTGTCG |
| *GPR125* | CCTTTCGGGGAATTTGTTTT | CCCGTACCGTGATGTTCTTC |
| *PIWIL2* | AGGACCATCAAGCTGTCACC | TTCATTACCCGACGGAAAAC |
| *ACR* | AACTCTGCGACAGAGGGAAA | ACACAAGTCCAGGTCGATGA |
| *POU5F1* | GAAGTTAGGTGGGCAGCTTG | TGTGGCCCCAAGGAATAGT |
| *NANOG* | GAGATGCCTCACACGGAGAC | AGGGCTGTCCTGAATAAGCA |
| *ID4* | TCCCGCCCAACAAGAAAG | TGTCGCCCTGCTTGTTCA |
| *NANOS3* | AAGGCGAAGACACAGGACAC | CGAAGGCTCAGACTTCCC |
| *c-Kit* | TAAGGCACTTTCTGGGCAGT | CTGCCAACCCCAGTAATGAT |
| *FGF1* | CCTGCAGGCTAGAGAAGCA | GATGCACTTTTTGCCCTTCT |
| *BLIMP1* | TAAAGCAACCGAGCACTGAGA | ACGGTAGAGGTCCTTTCCTTT |
| *CTGF* | CCTGCAGGCTAGAGAAGCA | GATGCACTTTTTGCCCTTCT |
| *CCND1* | GCTGTGCATCTACACCGACA | TTGAGCTTGTTCACCAGGAG |
| *CYR61* | AAACCCGGATTTGTGAGGT | GCTGCATTTCTTGCCCTTT |
| *MYC* | CCTCCCTCCACTCGGAAG | TCTGACACTGTCCAACTTGACC |
| *GAPDH* | GTCAACGGATTTGGTCGTATTG | CATGGGTGGAATCATATTGGAA |
|  |  |  |
| **List of antibodies for Immunofluorescent, Flow and Western blot** | | |
| **Antibody** | **Dilution/ Concentration** | **Vendor** |
| VASA or DDX4 | Western blot: 1:10000 | Abcam Catalog #: ab13840 |
| VASA or DDX4 | Flow cytometry: 1:200/10^5^ cells | Cell signaling Catalog #: 8761 |
| PLZF | Immunofluorescent: 8-25 µg/mL | R&D Catalog #: MAB2944 |
|  | Flow cytometry: 2.5 µg/10^6^ cells |  |
|  | Western blot: 1 µg/mL |  |
| PLZF Alexa Fluor 700-conjugated antibody | Flow cytometry: 0.25-1 µg/10^6^ cells | R&D Catalog #: IC2944N |
| FITC anti-human CD90 (Thy1) antibody | Flow cytometry: 5ul/10^6^ cells | Biolegend Catalog #: 328108 |
| APC anti-human CD117 (c-Kit) antibody | Flow cytometry: 5ul/10^6^ cells | Biolegend Catalog #: 313206 |
| CD90 or THY1 | Immunofluorescent: 1-100 | Abcam Catalog #: ab23894 |
| GPR125 | Immunofluorescent: 1-100 | Abcam Catalog #: ab51705 |
| PIWIL1 | Immunofluorescent: 10 µg/ml | Abcam Catalog #: ab12337 |
| Acrosin | Immunofluorescent: 1:100 | Thermo Fisher Catalog #: PA5-99580 |
|  | Western blot: 1:10000 |  |
| TNP1 | Immunofluorescent: 1 µg/ml | Abcam Catalog #: ab73135 |
| YAP@Rabbit | Immunofluorescent: 1:50 | Cell signaling Catalog #: 14074 |
| YAP@Mouse | Western blot: 1:10000 | Cell signaling Catalog #: 12395 |
| YAP@Rabbit | Western blot: 1:10000 | Cell signaling Catalog #: 4912 |
| YAP/TAZ | Western blot: 1:10000 | Cell signaling Catalog #: 8418 |
| Phos YAP | Western blot: 1:10000 | Cell signaling Catalog #: 4911 |
| Alexa Fluor 488 Goat anti-rabbit IgG | 1:500 | Thermo Fisher Scientific,  Cat# A11008 |
| Alexa Fluor 488 Goat anti-mouse IgG | 1:500 | Thermo Fisher Scientific,  Cat# A11001 |
| Alexa Fluor 594 Goat anti-Rabbit IgG | 1:500 | Cell Signaling Technology Cat# 8889 |
| β-ACTIN | Western blot 1:10000 | Sigma-Aldrich Cat# A3854 |
|  |  |  |

**Supplementary table S2**

| **hESC** | **Crispr/Cas9** | **shRNA** | **YAP/Actin** | **Spermatogonia-derived from SSC differentiation** |
| --- | --- | --- | --- | --- |
| WT | N/A | N/A | 1.40 | + |
| YAP-KD |  |  | 0.5813 | +++ |
| YAP-DKD |  |  | 0.25 | No |
