# Supplementary material for: The dynamic expression of YAP is essential for the development of male germ cells derived from human embryonic stem cells
Source: Sci Rep. 2024 Jul 8;14:15732. doi: 10.1038/s41598-024-66852-x (PMC11231333; doi:10.1038/s41598-024-66852-x)
Supplement: Supplementary file 3 — Supplementary Legends. [file 41598_2024_66852_MOESM3_ESM.docx]

**Supplemental Information**

**Supplementary Table S1:** **List of primers and antibodies used in this study**

**Supplementary Table S2: Expression level of YAP in each hESC lines used in this experiment and its spermatogenic differentiation efficiency.**

**Supplemental figure S1:** Representative micrographs show immunofluorescent staining of hESC- day0 before differentiation of DDX4/VASA, PLZF, CD90, GPR125 and PIWIL1.

**Supplemental figure S2:** Representative micrographs show immunofluorescent staining of Acrosin in human sperm positive control and hESC-day 0 as a negative control. Representative micrographs show immunofluorescent staining of TNP1 in human sperm and hESC-day 0 as a negative control.

**Supplementary Figure S3**. RNA sequencing analysis of human testicular tissue samples [16]fsu. (A) Color-coordinated map of all major types of human testicular germ cells that belong to various stages of spermatogenic differentiation. (B-H) Expression pattern of various human spermatogenic markers and YAP1 during *in vivo* spermatogenesis. Orange indicates the highest level of RNA expression, whereas dark blue indicates a low or no level of expression. The plots represent the continuum of spermatogenesis, and the individual dots represent individual cells. (I) The Wilcox rank sum test shows expression levels of YAP in human male germ cells during in vivo spermatogenesis. ****P<0.0001, ns = not significant difference.

**Supplementary Figure S4:** Characteristic of the YAP knockdown hESCs. Representative micrographs show normal ESC morphologies of YAP-KD and YAP-DKD cells. Scale; first column: 200 μm, second column: 100 μm, third column :100 μm and forth column: 50 μm

**Supplemental Figure S5:** *In vitro* spermatogenic cell differentiation of YAP knockdown hESCs. Representative micrographs of differentiated cells on day 0 (hESCs), day 5, day 7, day 10, and day 12 in WT, YAP-KD and YAP-DKD cells. Scale top: 200 μm, middle: 100 μm, bottom: 100 μm and zoom areas: 20 μm.

**Supplementary Figure S6:** Flow cytometry analysis of PLZF^+^-hSSCs during the spermatogenic differentiation of WT and YAP-KD cells on day 5, day 10, and day 12 and secondary antibody staining as the control group. Data were collected from 6 technical replications.

**Supplementary Figure S7:**  Modulation of YAP during spermatogenic cell differentiation. (A) Morphology of WT hESCs, 20 μM DH-treated cells, and 20 μM LPA-treated cells for the same periods. Scale bar, left: 200μm, middle: 100μm, right: 100μm. (B) Western blot confirmed the expression of YAP protein in hESCs after treatment with small molecules. (C) Quantification of protein level confirmed a statistically significant decrease in the YAP protein in DH-treated cells compared to untreated and LPA-treated cells. (D) Gene expression analysis confirmed down-regulation of the YAP target genes, *CTGF* and *CCN D1,* in cells treated with DH and up-regulation of *CTGF, CCND1,* and *CYR61* in cells treated with LPA. (E) Western blot showed expression of the YAP and PLZF protein in spermatogenic cells derived from cells treated with DH or LPA. (F) Quantification of protein level confirmed downregulation of YAP in DH-treated cells at the early time of day 0-5, and upregulation of YAP on day 12 after spermatogenic differentiation in all groups. DH-ET showed statistically significant up-regulation of the PLZF protein compared to LPA-ET and untreated cells in group 2. **P<0.05*, ***P<0.01*, ****P<0.001*, *****P<0.0001*, and # determined the comparison within the group. The uncropped membranes associated with Figure S5 are shown in Figure S12.

**Supplementary Figure S8 (Associated with Figure 2)**: Full-uncropped gels from Western blot analysis (A) Uncropped gels associated with Figure 2A. The selected band of YAP and β-ACTIN protein is represented in red box. (B-D) Uncropped gels associated with Figure 2G. Selected band of YAP, PLZF and β-ACTIN protein represented in red box. (E) The uncropped gels represent the protein ladder in the red box.

**Supplementary Figure S9 (Associated with Figure 3)**: Full-uncropped gels from Western blot analysis (A-B) Uncropped gels associated with Figure 3A. The selected band of VASA and β-ACTIN protein is represented in red box. (C-D) Uncropped membranes associated with Figure 3C. The selected band of acrosin and β-ACTIN proteins is represented in red box.

**Supplemental Figure S10 (Associated with Figure 4)**: Full-uncropped membranes from Western blot analysis. (A-B) Uncropped gels associated with Figure 4A. The selected band of YAP and β-ACTIN proteins is represented in red box. (C-G) Uncropped gels associated with the expression of WT protein in Figure 4C. Selected band of YAP, PLZF, VASA, Acrosin and β-ACTIN proteins is represented in red box. (H-L) Uncropped gels associated with the expression of the YAP-KD protein in Figure 4C. Selected band of YAP, PLZF, VASA, Acrosin and β-ACTIN proteins is represented in red box. (M-Q) Uncropped gels associated with the expression of the YAP-DKD protein in Figure 4C. The selected band of YAP, PLZF, VASA, Acrosin, and β-ACTIN proteins is represented in red box. (R-W) Uncropped gels associated with Figure 4F. The selected band of Pro-Caspase3, Cleaved-caspase3 and β-ACTIN proteins is represented in red box.

**Supplemental Figure S11 (Associated with Figure 5)**: Full-uncropped gels from Western blot analysis. (A-C) Uncropped gels associated with Figure 5D. The selected band of YAP, PLZF and β-ACTIN proteins are represented in red box.

**Supplementary Figure S12 (Associated with Supplementary Figure S7)**: Full-uncropped gels from Western blot analysis. (A-B) Uncropped gels associated with the Supplementary Figure S5B. The selected band of YAP and β-ACTIN proteins is represented in red box. (C-E) Uncropped gels associated with Supplementary Figure S5E. The selected band of YAP, PLZF and β-ACTIN proteins is represented in red box.

**Supplemental Figure S13: Differential expression of germ cell marker during spermatogenic cell differentiation in WT and YAP-KD cells.** (A) Graphs show the expression levels of primordial germ cell markers, *POU5F1*, *NANOG* and *BLIMP1*, in WT cells compared to those of YAP-KD cells. (B) Graphs show the expression levels of spermatogonia stem cell markers, *ID4* and *PLZF*, in WT cells compared to those of YAP-KD cells. (C) Graphs show the expression levels of early differentiated spermatogonia markers, *NANOS3* and *GFRA1*, in WT cells compared to those of YAP-KD cells. (D) Graphs show the expression levels of differentiated spermatogonia markers, *KIT* and *DNMT1*, in WT cells compared to those of YAP-KD cells. Data are presented as mean ± SD of 3 experiments. ***P <0.01*, ****P <0.001*, *****P <0.0001*.
